# DeepsmirUD: Prediction of Regulatory Effects on microRNA Expression Mediated by Small Molecules Using Deep Learning

**DOI:** 10.3390/ijms24031878

**Published:** 2023-01-18

**Authors:** Jianfeng Sun, Jinlong Ru, Lorenzo Ramos-Mucci, Fei Qi, Zihao Chen, Suyuan Chen, Adam P. Cribbs, Li Deng, Xia Wang

**Affiliations:** 1 College of Animal Science and Technology, Northwest A&F University, Yangling 712100, China; 2 Botnar Research Centre, Nuffield Department of Orthopedics, Rheumatology and Musculoskeletal Sciences, University of Oxford, Oxford OX3 7LD, UK; 3 Institute of Virology, Helmholtz Centre Munich—German Research Center for Environmental Health, 85764 Neuherberg, Germany; 4 Chair of Prevention of Microbial Diseases, School of Life Sciences Weihenstephan, Technical University of Munich, 85354 Freising, Germany; 5 Institute of Genomics, School of Medicine, Huaqiao University, Xiamen 362021, China; 6 Department of Computational Biology for Drug Discovery, Biolife Biotechnology Ltd., Zhumadian 463200, China; 7 Leibniz-Institut für Analytische Wissenschaften–ISAS–e.V., Otto-Hahn-Str asse 6b, 44227 Dortmund, Germany; 8 Department of Molecular and Cellular Biology, University of Arizona, Tucson, AZ 85721, USA

**Keywords:** drug discovery, miRNAs, small molecule compounds, regulatory effect prediction, deep learning

## Abstract

Aberrant miRNA expression has been associated with a large number of human diseases. Therefore, targeting miRNAs to regulate their expression levels has become an important therapy against diseases that stem from the dysfunction of pathways regulated by miRNAs. In recent years, small molecules have demonstrated enormous potential as drugs to regulate miRNA expression (i.e., SM-miR). A clear understanding of the mechanism of action of small molecules on the upregulation and downregulation of miRNA expression allows precise diagnosis and treatment of oncogenic pathways. However, outside of a slow and costly process of experimental determination, computational strategies to assist this on an ad hoc basis have yet to be formulated. In this work, we developed, to the best of our knowledge, the first cross-platform prediction tool, DeepsmirUD, to infer small-molecule-mediated regulatory effects on miRNA expression (i.e., upregulation or downregulation). This method is powered by 12 cutting-edge deep-learning frameworks and achieved AUC values of 0.843/0.984 and AUCPR values of 0.866/0.992 on two independent test datasets. With a complementarily constructed network inference approach based on similarity, we report a significantly improved accuracy of 0.813 in determining the regulatory effects of nearly 650 associated SM-miR relations, each formed with either novel small molecule or novel miRNA. By further integrating miRNA–cancer relationships, we established a database of potential pharmaceutical drugs from 1343 small molecules for 107 cancer diseases to understand the drug mechanisms of action and offer novel insight into drug repositioning. Furthermore, we have employed DeepsmirUD to predict the regulatory effects of a large number of high-confidence associated SM-miR relations. Taken together, our method shows promise to accelerate the development of potential miRNA targets and small molecule drugs.

## 1. Introduction

microRNAs (miRNAs) are a class of noncoding RNAs of approximately 20nt in size [1], which have important post-transcriptional regulatory effects [2,3]. miRNAs are often known to downregulate the expression of genes by inhibiting translation or promoting the degradation of target mRNAs [4,5], thereby exerting an impact on the gene regulatory pathways to, for example, remodel bone homeostasis [6] or give rise to malignancies [7]. It has been reported that up to thousands of mRNAs can be targeted by a single miRNA [8], which highlights the key role of miRNAs in gene regulatory networks. Growing evidence has suggested that alterations in the expression of miRNAs can either lead to a variety of cancer diseases [9] or suppress tumor progression [10]. For instance, the suppression of apoptosis in *Myc*-induced lymphomas entails amplifying the miR-17/92 cluster, whereas the genetic ablation of the endogenous miR-17/92 allele can enhance apoptosis and reduce tumorigenicity. In this regard, therapeutics can be achieved by targeting oncogenic miRNAs with potential drug molecules for expression alterations [11,12,13,14]. In particular, many small molecules (SMs) are reported to hold great promise as drugs of miRNA targets [15,16,17,18].

Experimentally verified measures to determine whether binding relationships exist between small molecules and miRNAs (SM-miR) are normally time-consuming and costly [19,20] in that it is challenging to experimentally study all possible combinations with a given pool of SM and miRNA candidates. According to the SM2miR database, which was built using data from more than 2000 publications [21], only 1.14% of all possible SM-miR pairs interwoven with 1492 unique miRNAs and 212 unique small molecules (after pre-processing) have been experimentally verified. Therefore, improving computational techniques to speed up the inference of binding is in high demand. Over the past decade, approaches for predicting the binding of small molecules to miRNAs have been widely studied, and the predictive power has been gradually sharpened [22,23,24,25]. This has led to methods developed based on similarity (e.g., SMMART [26]) or machine-learning inference techniques (e.g., PSRR [27]). Despite the tremendous efforts made in the binding inference, issues related to predicting the direction (i.e., downregulation or upregulation) of miRNA expression mediated by the binding small molecules have remained computationally unexplored. The computation-driven SM-miR regulation type-specific identification and analysis have important implications for speeding up the supply of direct or indirect evidence for miRNA-involved cancer pathogenesis and therapeutics.

Advances in deep learning have spawned ample opportunities to promote biological applications and discoveries [28,29,30], such as protein structural [31] and functional [32] prediction. In order to maximize method performance, we sought a variety of convolutional neural network (CNN)- and recurrent neural network (RNN)-based models from the computer-vision and speech-recognition fields [33,34]. These models, the majority of which can be trained quickly due to the residual connection in design [35] or required parameter numbers, allow relatively full-scale examination and comparison of performance, from shallow to ultradeep layers, visually and semantically.

Here, we present a deep-learning niche comprising 12 frameworks to predict SM-mediated regulatory effects upon miRNA expression. A comprehensive analysis was made to opt for those deep learning models optimized sufficiently and properly based on curated SM-miR relations and their biophysical and biochemical features. By avoiding overtraining rigorously, the fusion model of a long short-term memory (LSTM) neural network and a CNN (LSTMCNN) [36,37] is reported to outperform the rest of the models on experimentally resolved relations, and ResNet-based models [35], including ResNet18, ResNet50, and SCAResNet18 [38], are preferable to attain the most stable prediction performance over long training epochs. After achieving AUC values of 0.80–0.92 by using individual models, the final ensemble model, DeepsmirUD, can tap into their variance-reduced predictions to obtain a further boosted performance gain of up to ~2% in AUC and ~1–2% in AUCPR. By trawling through miRNA-disease data from miRCancer [39], we finally established a database of SM–cancer associations to provide potential drugs based on the SM-miR upregulation and downregulation profiles predicted by DeepsmirUD.

## 2. Results

### 2.1. Model Determination Using Full-Scale Stabilities in Predicting Regulatory Effects

**Figure 1:**
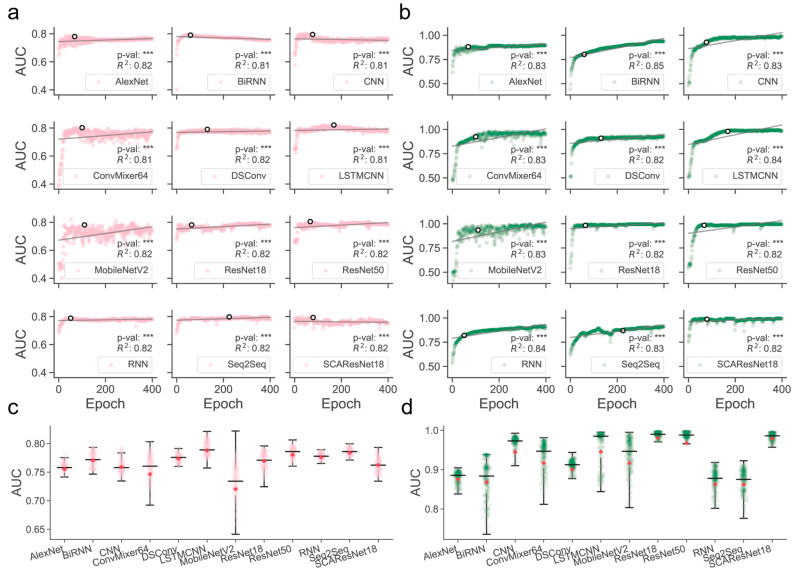
Overview of the full-scale performance examination of deep learning algorithms on independent test SM-miR relations over training epochs. Landscapes of AUC variations on Test (**a**) and TestSim (**b**). Boxplots of AUC values on Test (**c**) and TestSim (**d**). The *p*-val symbols show the statistical significance based on the T-test and *** represents the statistically significant difference. *R*^2^ represents r-squared values. The black hollow circles represent the final models used in DeepsmirUD. The red dots in the boxplots represent the average prediction values. Note that based on the early stopping strategy, the models were determined according to the performance reported on the Test dataset (**a**) at epochs 68, 59, 77, 100, 131, 168, 110, 64, 67, 51, 225, and 79 for AlexNet, BiRNN, CNN, ConvMixer64, DSConv, LSTMCNN, MobileNetV2, ResNet18, ResNet50, RNN, Seq2Seq, and SCAResNet18, respectively.

Determination of those models that are better trained over epochs is one of the major precursors of maximizing predictive performance when the models are optimized on a dataset of finite size. Often, limited by the size of collected samples, the models do not necessarily have to perform ideally for all kinds of test datasets. However, if quality control of the models during or after training was poorly conducted, their generalization abilities for different kinds of test data could become substantially worse, thereby likely losing the magnitude of the models for use in real-world applications. It was shown, for example, in residue contact prediction, that the performance of various models could rise ideally or grossly slip when they were examined on different purpose-built datasets [40,41]. In order to obtain models with a better generalization ability, we enlarged the size of the data required for training, mixed the SM-miR relations from different sources, employed a wide array of deep learning algorithms (see detailed descriptions in Section 4.6), and fully monitored performance variations via various assessment metrics with a large epoch number (Figure 1). We found that apart from MobileNetV2, the rest of the deep learning approaches mostly present a quite stable performance variation on the ebb and flow basis across 400 epochs. After reaching an AUC value of 0.8 in dozens of epochs, around half of all the methods leveled off, as evidenced by the overall profiles, statistically significant *p*-values, and R-square coefficients (i.e., >0.8). In combinations of the AUC and AUCPR profiles, we found that ResNet-wise methods, including ResNet18, ResNet50, and SCAResNet18, are more prone to obtain the most stable performance, while MobileNetV2 and ConvMixer64 produce models that oscillate between AUC values of 0.6 and 0.8 by a large margin on the Test dataset (Figure 1a,c). In addition, BiRNN shows a decrease in AUC over the epochs. Overall, the majority of the deep learning approaches have steady performance, which provides proof that the used features could be better acquired by the deep learning frameworks. The best models for each method were finally determined by the early stopping strategy. Moreover, the AUCPR, ACC, bACC, precision, recall, F1-score, MCC, and Jaccard values (changing with epochs on the Test and TestSim datasets) can be found in Supplementary Figures S2–S17.

### 2.2. Performance of SM-Mediated Regulatory Effects on miRNA Expression Using an Independent Dataset

**Figure 2:**
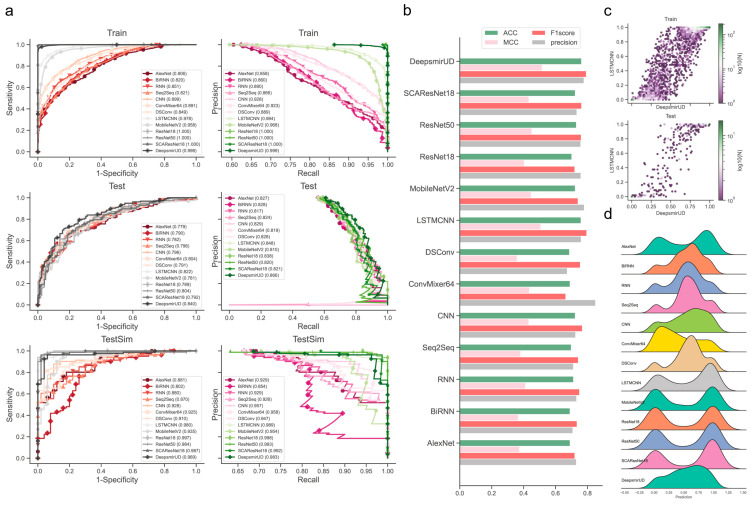
Performance evaluation of the resulting deep learning models on independent test SM-miR relations. (**a**) ROC and precision-recall (PR) curves on the Train, Test, and TestSim datasets. (**b**) ACC, MCC, F1 score, and precision on Test. (**c**) Hexagonal-binned plot of a comparison between predictions of the two leading models (LSTMCNN and DeepsmirUD) on Test. (**d**) Ridge plot of prediction values (i.e., regulatory effects) on Test.

We observed that the individual models determined using the above procedures exhibited good performance in predicting regulatory effects based on the Train (Supplementary Figure S18a and Table S1) and Test (Figure 2 and Table 1) datasets. Among them, LSTMCNN shows the best AUC and AUCPR performance. As a result of integrating the top-ranked best-performing models or all the 12 models, the two ensemble models, DeepsmirUD-top and DeepsmirUD-all, outperformed the individual models, with an AUC value 0.840/0.843 and an AUCPR value 0.866/0.866. All the models achieved an AUC value of above 0.770 and an AUCPR value of above 0.810. The ResNet-based methods gave a prediction distribution with two even peaks for the downregulation and upregulation classes, while the RNN-based methods yielded prediction values floating around 0.5 (Figure 2c,d). In addition, ConvMixer64, one of the most recent Vision Transformer (ViT) methods [42], is prone to producing downregulation-type predictions and obtains the best precision of over 0.8 (Figure 2b,d). Almost all methods have an insignificant difference in ACC and F1-score.

### 2.3. Regulatory Effect Prediction on Guilt-by-Association SM-miR Relations

Pairwise combinations of all unique miRNAs and small molecules derived from experimentally verified SM-miR relations can create a new SM-miR relation space in fathomless size. Apart from the known SM-miR relations, this space can contain massive potential upregulation and downregulation relations. In order to gain an ability to predict such potential upregulation and downregulation relations, we trained our models on samples mixed with SM-miR relations, which were formed using guilt by association (introduced in Section 4.2). Then, we tested them on the TestSim dataset. Surprisingly, all models, especially DeepsmirUD-top and ResNet18 achieved extremely high predictive performance with AUC and AUCPR values of up to 1 (Figure 2a, Supplementary Table S2 and Figures S17–S19). BiRNN performed the worst compared to any of the other methods. This suggests that most of our deep learning algorithms can also capture the characterization of the guilt-by-association-derived SM-miR relations of upregulation and downregulation.

### 2.4. Regulatory Effect Prediction Using Recurrent miRNAs or Small-molecule compounds

**Figure 3:**
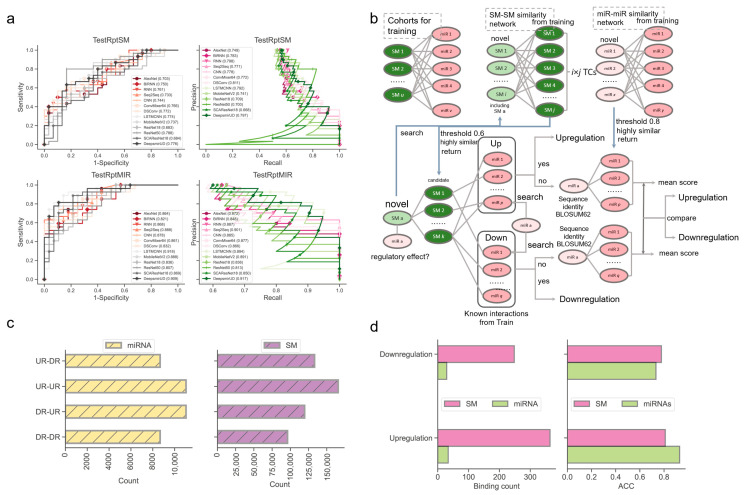
Performance evaluation using the TestRptSM, TestRptMIR, TestUniqSM, and TestUniqMIR datasets. (**a**) ROC and PR curves of deep learning algorithms on the TestRptSM and TestRptMIR datasets. (**b**) Similarity-based network inference of regulatory effects of SM-miR relations formed with novel small molecules or miRNAs. (**c**) Counts of combinations of novel miRNAs/small molecules (from upregulation/downregulation relations) and training miRNAs/small molecules (from upregulation/downregulation relations). The counts reflect the capacity of the two kinds of similarity networks in (**b**) (upper). (**d**) Bar plots of downregulation and upregulation binding counts and ACC.

Next, we examined the ability of our methods to predict the regulatory effects of SM-miR relations, where their miRNAs or small molecules appeared at least once in the training SM-miR relations (Figure 3a). Using TestRptSM, DeepsmirUD-top achieved the best performance, with AUC and AUCPR values of 0.807 and 0.814. Other top-ranked best-performing methods included DeepsmirUD-all, LSTMCNN, DSConv, and ConvMixer64, achieving AUC and AUCPR values in the vicinity of 0.770 and 0.790 (Supplementary Tables S3 and S4). Using TestRptMir, DeepsmirUD-top remained the best, with AUC and AUCPR values of 0.930 and 0.932, followed by DeepsmirUD-all and LSTMCNN, with AUC values of 0.909 and 0.918 and AUCPR values 0.917 and 0.894. Overall, most of the deep learning algorithms achieved good performance using the recurrent miRNAs or small molecules, which may imply their acceptable power for screening small molecule drugs or miRNA targets in practice, considering that the training dataset included various widely-used small molecules and miRNAs.

### 2.5. Regulatory Effect Prediction Using Novel miRNAs or Small-Molecule Compounds

We then explored how accurately our models were used to predict the regulatory effects of SM-miR relations, with each formed using either novel miRNA or novel small molecule. We observed that the model performance on TestUniqSM and TestUniqMIR was unsatisfactory and more susceptible to using novel small molecules than using novel miRNAs. To exclude sample bias, we altered random seeds 2–3 times to select the samples. But the results were similar to the observation above. In effect, almost all deep learning methods were incapable of accurately predicting the regulatory effects when the small molecules are novel but have a better AUC value of around 0.5 when the miRNAs are novel.

To offer a better predictive ability, we further constructed a similarity-based network inference approach to assist the optimization-based learning algorithms (described in Section 4.6, Figure 3b). Using the network inference approach and the relations formed with novel miRNAs, we obtained high ACC values of 0.929 and 0.733 in regard to predicting the upregulation and downregulation relations. Using the relations formed with novel small molecules, we achieved ACC values of 0.810 and 0.778 (0.813 on average, Figure 3d). It should be noted that this network approach does not always return prediction results (i.e., upregulation or downregulation) because the inference process of using the miRNA–miRNA, SM–SM, and/or SM–miRNA networks can be aborted at any step where no or not enough miRNAs/small molecules in the networks are similar to the input miRNAs/small molecules.

### 2.6. Reference Map of the Relations Based on Gene-Expression-Perturbed miRNAs or Small Molecules

The Psmir database employed an enrichment-score (ES) technique for determining the associations of a large number of SM-miR pairs and measuring their SM-miR responses (similar to the regulatory effects). We applied our deep learning methods to infer the upregulation and downregulation types of these associated SM-miR relations derived from Psmir in an effort to expand the knowledge of the unexplored SM-miR relations (Supplementary Tables S5 and S6). Our results demonstrated that the DeepsmirUD-determined upregulation and downregulation profiles are, to some extent, in agreement with Psmir’s (*p*-value ≤ 0.01: Supplementary Tables S7–S14; see also Supplementary Results). We present two examples using FDA-approved small molecules on DeepsmirUD-Web (chord diagrams): one for the predicted downregulation relations overlapped with Train in terms of miRNAs and the other one for the predicted upregulation nonoverlapped relations with Train in terms of miRNAs.

### 2.7. Prediction of Regulatory Effects for Indirect SM-miR Relations

**Figure 4:**
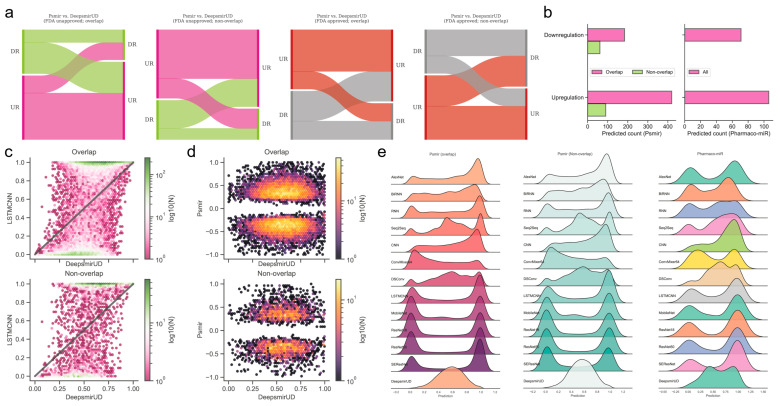
Reference map construction of regulatory effects for the SM-miR relations from Psmir and VerSe. (**a**) Alluvial diagram of the predicted regulatory effects for the FDA-unapproved and FDA-approved relations with the miRNAs/small molecules that are overlapped and non-overlapped with miRNAs/small molecules in Train. (**b**) Prediction of upregulated and downregulated SM-miR relations from Psmir and VerSe using DeepsmirUD. Hexagonal-binned plots show a comparison of the predicted regulatory effects between DeepsmirUD and LSTMCNN (**c**), and DeepsmirUD and Psmir (**d**). (**e**) Ridge plots of the predicted regulatory effects.

Using the deep learning methods, we further predicted the regulatory effects of 224 indirectly linked SM-miR relations, which were screened using miRNA pharmacogenomic data (Supplementary Table S15), and found that around two-thirds of SM-miR relations were predicted as upregulating (Figure 4b,e). As an example, we present the predicted upregulation relations on DeepsmirUD-Web (chord diagrams). All predictions are also available on DeepsmirUD-Web.

### 2.8. Inference of SM-Disease Associations for Drug Discovery and Repositioning

**Figure 5:**
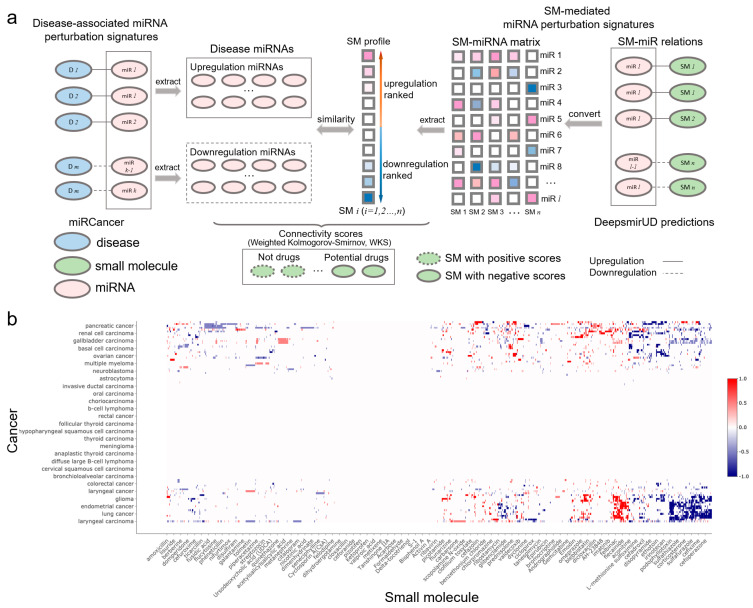
SM-cancer connectivity analysis. (**a**) Illustration of connectivity scoring to infer SM–cancer associations. (**b**) Heatmap of connectivity between 1343 small molecules and 107 cancer types. The positive connectivity score in red represents similar perturbations between a disease signature and a drug profile, while the negative connectivity score in blue stands for the drug reversal of the disease signature. Note that a well-resolved version of the SM–cancer heatmap can be found on DeepsmirUD-Web.

Small molecules that are able to rectify the abnormalities of the miRNA perturbation profiles in diseases can potentially be used for disease treatment, which provides insight into the drug mechanism of action. By utilizing miRNA-cancer associations from the miRCancer database and SM-miR relations predicted by DeepsmirUD, we generated SM–disease relationships for drug discovery and repositioning [25,43,44]. This demonstrates the ability of small molecules to alter miRNA perturbation profiles in cancers. To evaluate the extent to which a small molecule can treat a cancer disease, we calculated the connectivity scores based on similarity between SM-mediated miRNA perturbation profiles and cancer-associated miRNA perturbation profiles (Figure 5b). The final predicted associations involve a total of 107 cancers and 1343 small molecules, which are available at https://aidrugud.github.io/deepsmirud (accessed on 28 September 2022). A negative score suggests the pharmaceutical potential of a small molecule to treat a cancer disease, while a positive score suggests a similar perturbation profile between a small molecule and a cancer disease.

We present two case studies in which the identified drug-like small molecules have tangible anticancer effects, as validated by other studies. The FDA-approved antibiotic sulfafurazole has been found to inhibit tumor growth and metastasis in breast cancer by targeting the endothelin receptor A [45]. Meticrane has been verified to suppress squamous cell carcinoma [46]. Furthermore, we found that many top-ranked associations of single, small molecules targeting multiple cancers were predicted correctly, which is in line with previous studies. For example, cervical cancer, lung cancer, and colon cancer can be inhibited by naringin [47], which is validated by our calculated negative connectivity scores.

## 3. Discussion

In this paper, we have presented a novel deep learning tool to quantify small-molecule-mediated regulatory effects on miRNA expression (i.e., an up- or downregulation type) by training 12 deep learning architectures together as an ensemble: DeepsmirUD. The biological features that can reflect down- or upregulation signatures remain intact after being converted to image-like objects or acoustic signals. These features are then acquired visually and acoustically by image and speech recognition methods correspondingly. The inferred regulatory effects reflect the confidence of how strongly miRNAs are upregulated or downregulated by small molecules. DeepsmirUD can be applied for computationally screening miRNA targets or small molecule drugs on a large scale, using a given set of predicted or experimentally-verified associated SM-miR relations. In summary, our methods are expected to speed up the development of therapeutics to treat the disease-associated pathways that miRNA targets regulate.

Accumulated pharmaceutical studies released in recent years might be enriched for evidenced SM-miR relations. There is, doubtless, a dire need for establishing a publication-based database as an extensive repertoire of SM-miR relations. This will possibly be beneficial to further improve the performance of machine learning methods by getting rid of underfitting. Thus, the database establishment will be a promising direction of the future work. In addition, it will be interesting to predict the small molecule-mediated regulatory effects on the expression of other types of noncoding RNAs (e.g., siRNAs and lncRNAs).

## 4. Materials and Methods

### 4.1. Experimentally Verified SM-miR Relations

Similar to existing studies, we derived the SM-miR relations from the SM2miR database [21]. After removing corrupted data (e.g., repeated entries found from different publications) from 4989 entries in SM2miR, we first obtained 3641 SM-miR relations, with 2067 upregulation and 1574 downregulation relations. Next, we removed those relations with miRNAs of no FASTA sequences and/or small molecules of no SMILE strings. To retrieve the molecular sequences of miRNAs, we downloaded the miRbase database (version 22.1) [48]. To fetch the SMILE strings of small molecules from PubChem [49], we utilized their compound identities (CIDs) using PubChemPy (https://github.com/mcs07/PubChemPy (accessed on 11 April 2017)). All small molecules were confirmed to have certain SMILE strings with a total of 173 unique CIDs presented in the upregulation relation set and 153 unique CIDs presented in the downregulation relation set. After mapping our collected miRNA IDs to the miRbase IDs, we were left with 2037 upregulation relations with 1104 unique miRNAs of known sequences, and 1555 downregulation relations with 867 unique miRNAs of known sequences.

### 4.2. SM-miR Relations Based on Similarity Inference

The set of SM-miR pairs formed using all unique small molecules and miRNAs in SM2miR consists of both experimentally verified relations and unknown relations. If fully exploiting the unknown relations, we might be allowed to identify more of those that are potentially upregulation- and downregulation associated, which further increase the size of samples for deep learning and possibly enhance the generalization abilities of models due to a diverse composition in training samples. A popular approach to allow for inferring the potential binary relations is guilt by association [50,51], which deduces upregulation and downregulation relations based on the similarities of drugs and targets. Similarities between any two miRNAs were measured by sequence identities using the Pairwise2 module in Biopython [52] based on the BLOSUM62 matrices of two sequences (detailed in [53]). Due to restriction of the Pairwise2 module being applied only for DNA sequences, we replaced uracil in RNA sequences with thymine ahead of computing the sequence identities. Similarities between two small molecules were measured using Tanimoto coefficients [54] of their Morgan fingerprints, calculated by RDKit [55]. Any two small molecules are identified similar if their Tanimoto coefficient is greater than 0.6, as used in [54], and dissimilar, otherwise. Given that miRNA sequences are generally around 20 bases long, we loosened the threshold for gauging the similarity and dissimilarity. Any two miRNAs were identified similar if their sequence identity is above 0.8 (discussed further in Section 2) and dissimilar, otherwise.

**Figure 6:**
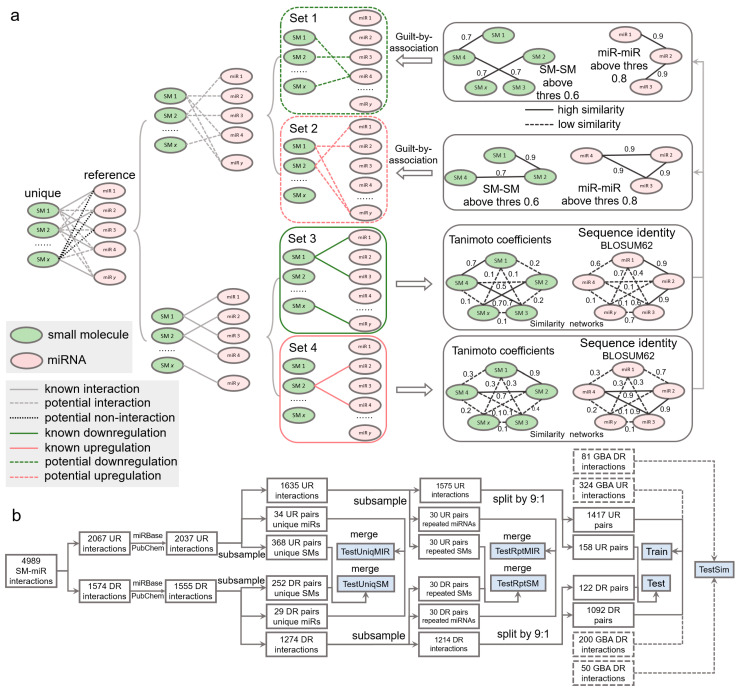
Data wrangling of upregulation and downregulation relations. (**a**) Inference of potential upregulation and downregulation relations using guilt by association. (**b**) Flowchart of generating the Train, Test, TestSim, TestRptMIR, TestRptSM, TestUniqMIR, and TestUniqSM datasets. UR: upregulation. DR: downregulation. GBA: guilt by association. SM: small molecule. miR: miRNA.

Inference of whether small molecules and miRNAs are potentially associated is performed by guilt by association in a way that associated and nonassociated SM-miR relations are screened from unknown SM-miR relations by a similarity-based comparison with known associated SM-miR relations. In this process, the guilt-by-association approach is applied only once, which is able to obtain the potentially associated or nonassociated SM-miR relations [54]. In contrast to such inference, there are three relationships in unknown relations that need to be considered to screen potential upregulation and downregulation relations: (1) nonassociated, (2) upregulation associated, and (3) downregulation associated. To bypass selections that could be erroneously made from the nonassociated relations, we screened the potential upregulation and downregulation relations separately by applying the guilt-by-association approach twice, as shown in Figure 6a. Fully-connected networks composed of sequence identities between unique miRNAs derived from experimentally verified upregulation and downregulation relations were generated separately, and so were those of the unique small molecules. In total, 608,856 miRNA-miRNA sequence identities and 14,878 SM-SM Tanimoto coefficients were calculated to infer the potential upregulation relations, while 375,411 miRNA-miRNA sequence identities and 11,628 SM-SM Tanimoto coefficients were used to infer the potential downregulation relations. Consequently, using a Tanimoto coefficient threshold of 0.6 and a sequence identity threshold of 0.8, we generated 405 and 250 potential upregulation and downregulation relations, respectively.

### 4.3. Dataset

To comprehensively investigate the performance of our deep learning tools, we constructed a number of test datasets (Figure 6b). To understand whether our tools gain a generalization ability in regulatory effect inference, from the aforementioned 2037 upregulation relations, we first randomly extracted 34 and 368 relations containing 20 unique miRNAs and 20 unique small molecules, respectively. Similarly, we obtained 29 and 252 relations from the 1555 downregulation relations. The 34 and 29 relations were combined to be used in the TestUniqMIR set, and the 368 and 252 relations were finally present in the TestUniqSM set. To test the performance of how sufficiently the deep learning approaches were trained, from 1635 remaining upregulation relations, we next randomly collected 30 relations (set A), with miRNAs appearing in the remaining relations at least once, as well as 30 relations (set B) with small molecules appearing in the remaining relations at least once. The same procedures were repeated to perform another miRNA-orientated pick of 30 relations (set C) and a SM-orientated pick of 30 relations (set D) from 1274 remaining downregulation relations. Set A and set C were combined as TestRptMIR, and Set B and set D were combined as TestRptSM. In a ratio of 9:1, we randomly split the left 1575 upregulation and 1214 downregulation relations for training and testing.

To enlarge the scale of the training data and further augment the data, we curated SM-miR relations from a space of unknown relations formed with all small molecules and miRNAs appearing in the datasets above. To avoid a lopsided ratio between the upregulation and downregulation training samples, the aforementioned 405 and 250 potential upregulation/downregulation relations were randomly split in a ratio of 4:1 for training and testing. Taken together, we generated a total of 3033 (1471 + 1092 + 324 + 200) training samples (Train), and 411 (158 + 122 + 81 + 50) test samples (Test). To ensure no overlaps between the training and test samples due to the addition of the guilt-by-association relations for training, we finally detected and removed 2, 5, and 7 overlapped relations from Test, TestRptMIR, and TestUniqSM, respectively. Detailed information about the datasets can be found in Supplementary Tables S16–S23.

In a word, by constructing TestRptMIR and TestRptSM, we can examine the predictive ability of DeepsmirUD on a new relation with its miRNA or its small molecule that DeepsmirUD has seen during training, respectively, while by constructing TestUniqSM and TestUniqSM, we can examine the predictive ability on a new relation with its miRNA or its small molecule that DeepsmirUD has never seen during training, respectively. Besides, TestSim is constructed to examine the performance of the guilt-by-association SM-miR relations. The test performance using these datasets can allow us to gain a better understanding of the fitness of DeepsmirUD in multiple application scenarios.

### 4.4. Definition of the Small-Molecule-Mediated Regulatory Effects on miRNA Expression

In this work, the small-molecule-mediated regulatory effect on miRNA expression refers to the likelihood of the downregulation or upregulation of an associated SM-miR pair, i.e., how likely the expression of a miRNA is downregulated or upregulated by a small molecule.

Mounting attention (of the current computational techniques) is being paid to predicting the associations between small molecules and miRNAs. As shown in Figure S1, DeepsmirUD, in essence, distinguishes itself from others by a sequentially ordered regulatory effect determination process on completion of association determination. To be more exact, in this pipeline, the association determination step yields whether and how likely an association between a SM-miR pair exists, yet it remains unknown as to which regulation type (i.e., upregulation or downregulation) they have. Subsequently, the pair having been experimentally evidenced or predicted as being associated will be passed on to DeepsmirUD for predicting its regulation type (i.e., regulatory effects).

### 4.5. Feature Representation

**Figure 7:**
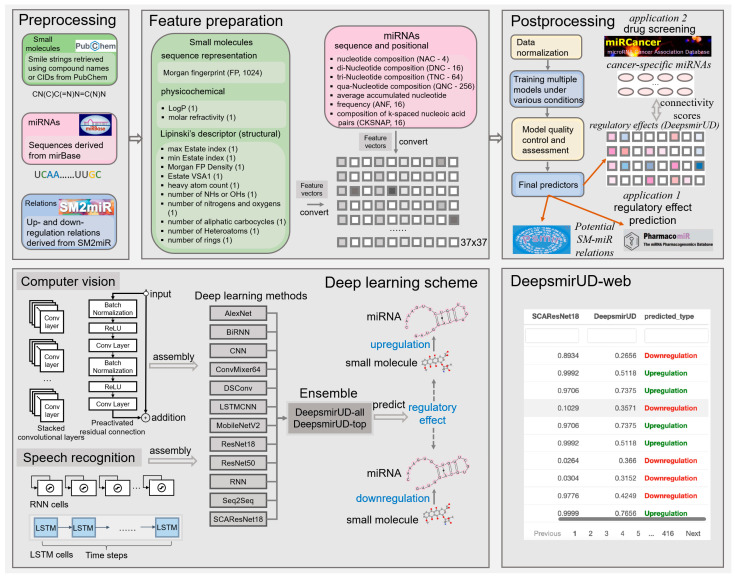
Workflow of predicting the SM-mediated regulatory effects on miRNA expression by deep learning algorithms. In box Feature preparation, the integers stand for the length of the features. The feature vector stands for the concatenation of the miRNA and small-molecule features.

We make use of a 1396-size feature vector to characterize the upregulation or downregulation relation between a small molecule and a miRNA (Figure 7). It reflects the positional, compositional, physicochemical, or structural properties of a small molecule or a miRNA, with the former represented by a vector length of 360 and the latter represented by a vector length of 1036. Given that the feature extraction is performed on an image-like input by the computer-vision methods, including AlexNet, CNN, ConvMixer, DSConv, LSTMCNN, MobileNetV2, ResNet, and SCAResNet18 (see Section 4.6 for details), we then converted each feature vector to a 37 × 37 matrix with an initial channel equal to one, while at each time step, we sequentially cropped out 37 features from the feature vector, which were then taken as input into the speech-recognition methods, including RNN, BiRNN, and Seq2Seq. The features of the small molecules and miRNAs are listed below.

The miRNA vector is comprised of positional and compositional features.

Nucleotide Composition (NAC). NAC measures the percentage of a single nucleic acid type in a nucleotide sequence, which is computed by

$$NAC=\frac{N_{i}}{L}$$

where L represents the length of a miRNA sequence and $N_{i}$ represents the total count of a nucleotide i in the sequence.

Di-Nucleotide Composition (DNC). DNC measures the percentage of the combination of any two nucleotides in a nucleotide sequence, given by

$$DNC=\frac{N_{i,j}}{L-1}$$

where $N_{i,j}$ represents the total count of a pair of nucleotides, i and j, in the sequence.

Tri-Nucleotide Composition (TNC). TNC is used to describe the composition of 64 unique triplets of nucleotides for a sequence, which is expressed as

$$TNC=\frac{N_{i,j,k}}{L-2}$$

where $N_{i,j,k}$ represents the total count of a triplet of nucleotides, i, j, and k, in the sequence.

Qua-Nucleotide Composition (QNC). TNC is used to describe the composition of 256 unique quadruplets of nucleotides for a sequence, which is expressed as

$$QNC=\frac{N_{i,j,k,l}}{L-3}$$

where $N_{i,j,k,l}$ represents the total count of nucleotides i, j, k, and l in the sequence.

Composition of t-Spaced Nucleic Acid Pairs (CTSNAP). CTSNAP [56] is a 16-size vector containing the percentages of 16 possible pairs of nucleotides with a t distance apart, which are defined as

$$\mathrm{CTSNAP}=\frac{N_{i,j}^{t}}{L-t+1}$$

where $N_{i,j}^{t}$ represents the total count of a pair of two nucleotides (i,j∈AA, AC, AG, AT, CA, CC, CG, CT, GA, GC, GG, GT, TA, TC, TG, TT) in distance *t*.

Average Accumulated Nucleotide Frequency (aveANF). We define aveANF, which encodes both the positional and compositional information for a given nucleotide i, given by

$$aveANF=\frac{\sum_{n=1}^{N_{i}}\frac{\sum_{m=1}^{p_{n}}\delta_{m}}{p_{n}}}{N_{i}}$$

where $p_{n}$ represents the position of the nucleotide i, and $\delta_{m}$ is the Kronecker symbol to indicate whether a nucleotide, i, is present at position m.

$$\{\begin{array}{c} 0,\;\delta_{m}\neq i\\ 1,\;\delta_{m}=i \end{array}$$

Fingerprint. The Morgan fingerprint (length 1024) and the density of the Morgan fingerprint were encoded for small molecules.

Structural and physiochemical encoding. From each small molecule, we extracted 6 structural elements, including the number of rings, the number of heavy atoms, the number of nitrogens and oxygens, the number of NHs or OHs, the number of aliphatic carbocycles, and the number of heteroatoms. Besides, the maximum and minimum of the estate indexes and the Waals surface area combining estate were considered. Finally, LogP and molar refractivity, reflecting the compound’s physiochemical properties, were also encoded.

All miRNA features are implemented based on Python, and all small molecule features are generated using RDKit.

### 4.6. Deep Learning Methodology

It is well known that in recent years, the number of off-the-shelf deep learning models has gone into orbit [42] due to not only an increasing amount of input from research communities but also the flexibility in stacking or removing a number of unit neural network structures required for training and optimization, as well as changing other detailed settings within neural layers of note [57]. This is largely different from most of the traditional machine learning algorithms that rely on relatively fixed model structures. The performance of different deep learning models can also be slightly or largely different, which can be evidenced partially by a plethora of computer-science publications, with different models competing annually for better prediction abilities on standard training cohorts, such as CIFAR-10 [35]. Without a detailed benchmark, the choices of deep learning models are unknown. For this reason, we opted for 6 well-established deep learning models (Alexnet, ConvMixer, MobileNetV2, ResNet18, ResNet50, and SCAResNet18) and 6 self-assembled architectures (BiRNN, CNN, DSConv, LSTMCNN, RNN, and Seq2Seq) and comprehensively evaluated their capability of predicting SM-mediated regulatory effects on miRNA expression. The construction and training settings of the models are detailed below.

Alexnet. Alexnet was first introduced to perform an image recognition task in the LSVRC-2010 contest, which achieved state-of-the-art results [58]. The backbone of Alexnet consists of 5 convolutional layers and 3 fully-connected layers. We kept using the raw setting of Alexnet, with 96 filters (of size 11 × 11), 256 filters (of size 5 × 5), 384 filters (of size 3 × 3), 384 filters (of size 3 × 3), and 256 filters (of size 3 × 3) placed in the respective convolutional layers in order.

BiRNN. The bidirectional recurrent neural network (BiRNN) is a variant version of the recurrent neural network (RNN), which can be trained in forward and backward time directions simultaneously [59]. Our BiRNN structure consists of a single BiRNN layer preceded by two RNN layers, each with 256 hidden units.

CNN. As one of the most typical deep learning components, convolutional neural networks (CNNs) have been pervasively used in multiple kinds of fields [33]. We constructed a 3-layer CNN structure, each followed by a max pooling operation with a step of 2. Finally, a 128-neuron fully-connected layer was placed after completing all convolution operations. We applied 32 filters (of size 3 × 3) to extract features from input.

ConvMixer64. Very recently, the ConvMixer model has been proposed as a type of vision transformer (ViT) to handle image recognition by learning the patches of image-like objects [42]. The ViT module can remove the inductive biases of convolution operations to boost performance to some extent, thereby starting to be popularly applied in computer vision. We kept the backbone of the ConvMixer model the same as in [42] but to maintain the computing power for training at a manageable level, we replaced 256 filters with 64 filters for feature extraction and reduced the number of ConvMixer blocks from 8 to 2.

DSConv. In the Xception method, the recurrent depthwise-separable convolution (DSConv) modules are described as the cornerstone of high performance for image recognition [60,61]. The key of DSConv lies in a hypothesis that the full-scale separation between cross-channel correlations and spatial correlations in relation to the feature maps of CNN may have a positive impact on performance increase. This has been tested efficient to improve image recognition performance. We were interested in whether our study could, to some extent, benefit from the use of the DSConv operation alone, and thus constructed a DSConv-based deep learning framework consisting of solely the DSConv operation that alternates between a depthwise convolutional layer and a separable convolutional layer (2 times), which finally connects to a 128-neuron fully-connected layer. The max pooling operation with a size of 2 was performed only after a depthwise convolutional layer. Likewise, 32 filters (of size 3 × 3) were applied to extract features from input.

LSTMCNN. A LSTMCNN layer is a hybrid of a convolutional layer and a long short-term memory (LSTM) layer [34,36]. The output of the LSTM component is a convolution-like transformation that suits the input of the next convolutional layer. We constructed a structure with 3 LSTMCNN layers, each followed by a max pooling operation with a step of 2. We applied 32 filters (of size 3 × 3) to extract features from input. We used the ConvLSTM2D module of Tensorflow as each LSTMCNN layer.

MobileNetV2. MobileNetV2 was designed for object recognition tasks in mobile and resource-constrained environments and has been shown to effectively remove nonlinearities in layers, demonstrating high predictive performance [62]. We introduced the raw MobileNetV2 model into regulatory effect inference. To maintain the acceptable consumption of CPU and memory resources on every single convolutional layer, we kept the filter number no greater than 64.

ResNet18. Residual neural networks (ResNets) have gained popularity in deep learning applications and have recently achieved great success in protein structure prediction [31]. A well-tried ResNet with 18 residually-connected convolutional layers used in [35] was adopted as our ResNet18 method.

ResNet50. There are two studies in predicting residue contacts [40] and interaction sites [63] in transmembrane proteins, which have benefitted from the use of ResNets with massive layers (>35). Therefore, in addition to the 18-layer ResNet, we also applied a 50-layer ResNet (used in [35]) to extract features and learn representations of the SM-mediated regulatory effects on miRNA expression.

RNN. The recurrent neural network (RNN) is one of the basic structures of deep learning algorithms, which is used quite commonly in speech and language processing [34,64]. Our RNN structure begins with 2 LSTM-type RNN layers, each with 256 neurons inside. It finally connects to a fully-connected layer with 256 neurons.

Seq2Seq. The sequence-to-sequence method has been introduced in modeling natural language semantically and syntactically [65]. It encodes and decodes a sequence of input symbols (i.e., feature vectors) and has been tested effective in learning the representations of the input symbols [66]. At the core of our Seq2Seq structure are one RNN-like encoder and one RNN-like decoder, followed by a fully-connected layer with 256 neurons. The encoder has exactly the same structure as our BiRNN algorithm, and so does the decoder but with the 3 layers placed in reverse order.

SCAResNet18. The attention mechanism is brought up for recasting and refining intermediate feature maps locally so as to enhance the representational power of deep learning algorithms in the spatial and channel-wise directions, leading to two derivatives: the spatial and channel-wise attention (SCA) modules introduced by Woo et al. [38]. We adopted one of their reported models, CBAM-ResNet18 integrating ResNet18 with the SCA modules. We term it SCAResNet18 in our study.

### 4.7. Training Deep Learning Algorithms

As in the two previous studies [40,63], we picked the Adam method [67] for the optimization of all these deep learning algorithms with a learning rate $1\times 10^{3}$ and a batch size of 100. The parameters of the deep learning algorithms were estimated based on 5-fold cross-validation. Categorical cross entropy was introduced to measure if the ground-truth labels were different from the predicted SM-miR relations.

### 4.8. Overfitting Prevention

Overfitting [68] has a prominent impact on lowering the generalization abilities of intelligent models on unseen SM-miR relations, especially for those formed with small molecules and/or miRNAs to be largely distinguishable (in features) to every single small molecule and miRNA in the training samples. To avoid overfitting, we adopted the early stopping strategy (previously used in [63]) to pick out the sufficiently trained models in a way that the training had no sooner terminated than the prediction performance showed a falling tendency on test/validation data.

### 4.9. Ensemble of Deep Learning Models

The ensemble models contained in the final DeepsmirUD tool were obtained by averaging predictions produced by all or part of the individual deep-learning models. Such a technique has been widely used and primarily aims to reduce variations among predictions [40,63,69]. We prioritized the choices of only the best-performing individual models that were most suited for making ensemble models for different application scenarios, which we referred to as DeepsmirUD-top. For each application scenario, the number and the combination of individual models varied and were determined until the best performance was tested out. In the meantime, for each dataset, the performance of the ensemble model of all the 12 individual models was also compared, which we referred to as DeepsmirUD-all.

### 4.10. Performance Assessment

Our deep-learning approaches were evaluated by threshold-free measurements, including AUC and AUCPR [63], and a number of threshold-based measurements, including accuracy (ACC), balanced accuracy (bACC), precision, recall, F1-score, the Jaccard index, and the Matthews correlation coefficient (MCC).

$$ACC=\frac{TP+TN}{TP+TN+FP+FN}$$

$$bACC=\frac{1}{2}\left(\frac{TP}{TP+FN}+\frac{TN}{TN+FP}\right)$$

$$Precision=\frac{TP}{TP+FP}$$

$$Recall=\frac{TP}{TP+FN}$$

$$F1-score\;\left(F1\right)=\frac{2\times Precision\times Recall}{Precision+Recall}$$

$$MCC=\frac{TP\times TN-FP\times FN}{\sqrt{\left(TP+FP\right)\left(TP+FN\right)\left(TN+FP\right)\left(TN+FN\right)}}$$

$$\mathrm{Jaccard}\;\mathrm{index}=\frac{TP}{TP+FP+FN}$$

where TP (true positive), FP (false positive), TN (true negative) and FN (false negative) stand for the number of upregulation SM-miR pairs to be predicted correctly as upregulating, the number of downregulation SM-miR pairs to be predicted incorrectly as upregulating, the number of downregulation SM-miR pairs to be predicted correctly as downregulating, and the number of upregulation SM-miR pairs to be predicted incorrectly as downregulating, respectively. For the threshold-based methods, a threshold of 0.5 was taken to calculate TP, FP, TN, and FN.

### 4.11. Network Inference Approach for Novel SM Drugs and miRNA Targets Based on Similarity

We further proposed a network inference approach for improving the prediction of the regulation types of SM-miR relations, each formed with either novel small molecule or novel miRNA. Since the size of cohorts (~3000) for training was limited and the difference between the training and unseen SM chemical structures can be very high, the deep learning power might be impaired when unseen small molecules or unseen miRNAs were used. This approach was built by taking advantage of the training sample information in the following ways. First, we built two similarity networks, one storing SM–SM similarity scores between all unique small molecules and all training small molecules and the other one storing miRNA–miRNA similarity scores between all unique miRNAs and all training miRNAs (Figure 3b,c). For a predicted or experimentally-verified SM-miR relation, the network inference approach can select a small molecule or a miRNA as input. As an example, we assumed that a query SM-miR relation was formed with a novel small molecule, and we began by searching small molecule candidates from the SM–SM similarity network (if any small molecule in this network shared a predefined threshold of 0.6 with the query small molecule). Then, those relations containing the returned small molecule candidates were picked from both sets of known upregulation and known downregulation relations (from the Train dataset) and had their miRNAs matching the partner miRNA of the query small molecule. If a perfect match appeared in the known upregulation/downregulation relations, the query relation was correspondingly assigned upregulation/downregulation. Otherwise, two subnetworks containing similarity scores between the partner miRNA and the picked upregulated and downregulated miRNAs were extracted from the miRNA–miRNA similarity network, respectively. Finally, the similarity scores in each subnetwork were averaged, and the highest one decided whether the query relation was an upregulation or downregulation type. We repeated the same workflow for those relations that are formed with novel miRNAs, but we screened the candidate miRNAs based on a query miRNA from the miRNA–miRNA similarity network with a threshold of 0.8 because small-length miRNAs are more amenable to causing a lot of high scores of similarities between miRNAs than small molecules of long fingerprint length. This approach is also included in the DeepsmirUD tool.

### 4.12. Psmir Relations Based on miRNA-Perturbed Gene Expression Profiles

Psmir is an archive of high-confidence predicted SM-miR relations, which are selected from a curated collection of SM-miR candidate relations. These relations were constructed with miRNAs from miRNA-perturbed gene expression profiles in the GEO database and small molecules from small molecule-perturbed gene expression profiles in cmap [70]. With a pool of 51,051 candidate SM-miR relations formed with 1309 unique small molecules and 39 unique miRNAs, 6501 relations containing 1295 unique small molecules and 25 unique miRNAs were screened as being associated by applying a statistically significant *p*-value ≤ 0.05. We identified 1195 small molecules whose compound names were successfully matched using PubChemPy (https://pypi.org/project/PubChemPy (accessed on 11 April 2017)) with certain small strings. We removed those relations, which shared the same miRNA IDs but had different compound names that were converted into the same CIDs, leaving 5001 SM-miR relations. Upon removal of relations with miRNAs in no match to any records in miRBase, we finally obtained 4656 SM-miR relations, of which 4156 (overlapped) have their miRNAs appearing in our training relations and 500 (nonoverlapped) have their miRNAs unique to any miRNA in our training relations. We also examined a set of high-quality relations filtered with a *p*-value ≤ 0.1. The negative or positive response of drugs to miRNAs in Psmir was determined by a negative or positive association score (AS), respectively.

### 4.13. VerSe with Pharmacogenomic miRNAs

VerSe supplies the painstaking curation of miRNA pharmacogenomic sets that essentially embody miRNA-gene-drug triplets in a ternary relationship, in which miRNAs inhibit genes and further affect the response of drugs related to the genes [71]. We attempt to dig up the potential influence of miRNAs/drugs on drugs/miRNAs from 272 SM-miR indirect links in VerSe. Note that VerSe does not provide whether SM-miR links are upregulations or downregulation types and if small molecules and miRNAs are indirectly related. After examining the availability of miRNA sequences using miRbase, we were left with 257 SM-miR indirect links and after eliminating the links with drugs (of no SMILE strings), we finally obtained 224 SM-miR indirect links. Similar to the deduplication procedure used in Psmir, after removing those relations sharing the same miRNA IDs but with different compound names mapped onto the same CIDs, we finally retained 177 SM-miR indirect links.

### 4.14. miRNA-Cancer Database

A total of 9080 cancer-miRNA relations were obtained from the miRCancer database (version: 27 August 2019) [39], which were collected from 7288 papers. This database contains the information about the upregulation and downregulation of miRNAs in cancers.

### 4.15. Connectivity Scoring of SM-Cancer Associations

Druglike capabilities of small molecules in treating cancers are evaluated according to connectivity scores (ranging from −1 to 1), which are calculated with the weighted Kolmogorov–Smirnov (WKS) method (https://github.com/Jasonlinchina/RCSM (accessed on 2 August 2019)) [72]. A negative score of a small molecule represents its mediated miRNA perturbation signature opposite to that of a query disease and suggests its therapeutic potential as a drug against the disease, while a positive score suggests the similarity between the SM-mediated and the disease-associated miRNA perturbation signatures [44,73]. As seen in Figure 5a, for a given disease, we first retrieved all genes transcribing the disease-related miRNAs and split them into two sets: one being upregulated and the other one being downregulated in the disease. Then, we built a SM×miRNA matrix based on the DeepsmirUD-determined probabilities of SM-miR relations being upregulated/downregulated. In this matrix, each small molecule’s miRNA perturbation signature is represented by its mediated regulatory effects on the expression of all miRNAs used. Finally, the disease miRNA signatures and the ranked miRNA signatures for each small molecule were compared to yield a series of potential drugs with negative connectivity scores. Note that the SM×miRNA matrix was built by taking advantage of all SM-miR relations used in this study in order to comprehensively screen drugs.

## Figures and Tables

**Table 1:** Prediction performance evaluation on Test. DeepsmirUD-all is the ensemble model of 12 individual models, and DeepsmirUD-top is the ensemble model of the top-ranked best-performing individual models (see Section 4.9).

Method	AUC	AUCPR	ACC	bACC	Precision	Recall	MCC	F1score	Jaccard
AlexNet	0.779	0.827	0.691	0.688	0.730	0.712	0.374	0.721	0.563
BiRNN	0.790	0.828	0.691	0.681	0.708	0.763	0.367	0.735	0.580
RNN	0.782	0.817	0.712	0.704	0.732	0.769	0.412	0.750	0.600
Seq2Seq	0.796	0.824	0.698	0.687	0.712	0.776	0.381	0.742	0.590
CNN	0.796	0.829	0.723	0.710	0.726	0.814	0.432	0.767	0.623
ConvMixer64	0.804	0.819	0.691	0.711	**0.850**	0.545	0.436	0.664	0.497
DSConv	0.791	0.828	0.687	0.663	0.673	**0.859**	0.359	0.755	0.606
LSTMCNN	0.822	0.848	0.759	0.749	0.760	0.833	0.507	0.795	0.660
MobileNetV2	0.781	0.810	0.723	0.726	0.780	0.705	0.448	0.741	0.588
ResNet18	0.789	0.838	0.701	0.704	0.759	0.686	0.404	0.721	0.563
ResNet50	0.804	0.820	0.730	0.726	0.758	0.763	0.452	0.760	0.613
SCAResNet18	0.792	0.821	0.723	0.713	0.734	0.795	0.433	0.763	0.617
DeepsmirUD-all	0.840	**0.866**	0.763	0.756	0.778	0.808	0.516	0.792	0.656
DeepsmirUD-top	**0.843**	**0.866**	**0.781**	**0.779**	0.810	0.795	**0.556**	**0.803**	**0.670**

## Data Availability

All training, testing, and benchmarking datasets used throughout this research are publicly accessible and can be downloaded from https://aidrugud.github.io/deepsmirud (accessed on 28 September 2022). The DeepsmirUD software package is publicly available at https://github.com/2003100127/deepsmirud (accessed on 28 September 2022), which can run on multiple platforms (e.g., Docker). Predictions of regulatory effects on miRNA expression for a large number of SM-miR relations and cancer type-specific drugs identified by using SM-miR relation predictions are available at https://aidrugud.github.io/deepsmirud (accessed on 28 September 2022) and https://github.com/rujinlong/DeepsmirUD_web (accessed on 28 September 2022). A total of 15 methods, including DeepsmirUD-top, DeepsmirUD-all, the similarity-based network inference approach, and 12 individual deep learning models, are added to the final DeepsmirUD software package. We suggest that according to our in silico experiments, DeepsmirUD-top, DeepsmirUD-all, and LSTMCNN are generally the methods of choice used.

## Supplementary Materials

The following supporting information can be downloaded at: https://www.mdpi.com/article/10.3390/ijms24031878/s1. References [23,27,74,75,76] are cited in the supplementary materials.

## Abbreviations

SM: Small molecule; miRNA: microRNA; SM-miR: Small molecule and miRNA; CID: Compound identity; CMAP: Connectivity map; ES: Enrichment score; NAC: Nucleotide composition; DNC: Di-nucleotide composition; TNC: Tri-nucleotide composition; QNC: Qua-nucleotide composition; aveANF: Average accumulated nucleotide frequency; CTSNAP: Composition of t-spaced nucleoic acid pairs; CNN: Convolutional neural network; Seq2Seq: sequence-to-sequence; DSConv: Depthwise-separable convolution; LSTM: Long short-term memory; RNN: Recurrent neural network; SCA: Spatial and channel-wise attention; ViT: Vision transformer; BiRNN: Bidirectional recurrent neural network; ResNets: Residual neural networks; LSTMCNN: the fusion model of a LSTM neural network and a CNN; ACC: accuracy; bACC: balanced accuracy; MCC: Matthews correlation coefficient; TP: true positive; FP: false positive; TN: true negative; FN: false negative; WKS: weighted Kolmogorov–Smirnov.
