# Supplementary material for: DeepsmirUD: Prediction of Regulatory Effects on microRNA Expression Mediated by Small Molecules Using Deep Learning
Source: Int J Mol Sci. 2023 Jan 18;24(3):1878. doi: 10.3390/ijms24031878 (PMC9915273; doi:10.3390/ijms24031878)
Supplement: Supplementary file 1 [file ijms-24-01878-s001.zip › ijms-2094832-supplementary/Supplementary_Material/Supplementary_Material.pdf]

Supplementary Material

DeepsmirUD: Prediction of regulatory effects on microRNA expression mediated by small molecules using deep learning

Jianfeng Sun[1,2,†], Jinlong Ru[3,4,†], Lorenzo Ramos-Mucci[2], Fei Qi[5], Zihao Chen[6], Suyuan Chen[7], Adam P. Cribbs[2], Li Deng[3,4,*], Xia Wang[1,8,*]

[1] College of Animal Science and Technology, Northwest A&F University, Yangling, 712100, China
[2] Botnar Research Centre, Nuffield Department of Orthopedics, Rheumatology and Musculoskeletal Sciences, University of Oxford, OX3 7LD Oxford, United Kingdom
[3] Institute of Virology, Helmholtz Centre Munich – German Research Center for Environmental Health, 85764 Neuherberg, Germany
[4] Chair of Prevention of Microbial Diseases, School of Life Sciences Weihenstephan, Technical University of Munich, 85354 Freising, Germany
[5] Institute of Genomics, School of Medicine, Huaqiao University, 362021 Xiamen, China
[6] Department of Computational Biology for Drug Discovery, Biolife Biotechnology Ltd, Zhumadian, 463200, China
[7] Leibniz-Institut für Analytische Wissenschaften –ISAS– e.V., Otto-Hahn-Str asse 6b, 44227 Dortmund, Germany
[8] Department of Molecular and Cellular Biology, University of Arizona, AZ 85721 Tucson, USA
[†] These authors contributed equally to this work.
[#] Correspondence should be addressed to li.deng@helmholtz-muenchen.de and xiawang@nwafu.edu.cn.

Supplementary Results

Using a p-value of 0.01 to screen high-quality Psmir relations, the upregulated Psmir relations (FDA-unapproved, overlapped with Train; FDA-unapproved, non-overlapped with Train; FDA-approved, overlapped with Train) largely agree to upregulated relations predicted by DeepsmirUD, while there is the other way around for the downregulated Psmir relations (Figure 4a and 4d). The regulatory effects of the SM-miR associations provided in Psmir are predicted as SM-mediated upregulating for miRNAs by most of the deep learning methods (Figure 4e, two best-performing methods in Figure 4c). For FDA-approved drugs and a p-value of 0.01, we obtained 36 relations predicted as upregulating with 34 unique SMs and 3 miRNAs (non-overlapped), 45 relations predicted as downregulating with 39 unique SMs and 3 miRNAs (non-overlapped), 414 relations predicted as upregulating with 281 unique SMs and 20 miRNAs (overlapped), and 167 relations predicted as downregulating with 148 unique SMs and 20 miRNAs (overlapped). For FDA-unapproved drugs and a p-value of 0.01, we obtained 41 relations predicted as upregulating with 38 unique SMs and 3 miRNAs (non-overlapped), 42 relations predicted as downregulating with 38 unique SMs and 3 miRNAs (non-overlapped), 443 relations predicted as upregulating with 276 unique SMs and 20 miRNAs (overlapped), and 167 relations predicted as downregulating with 141 unique SMs and 20 miRNAs (overlapped).

Table S1. Prediction performance evaluation on Train. DeepsmirUD-all is the ensemble model of 12 individual models and DeepsmirUD-top is the ensemble model of the top best-performing individual models (see *Materials and methods*).

Method	AUC	AUCPR	ACC	bACC	Precision	Recall	MCC	F1score	Jaccard
AlexNet	0.806	0.858	0.714	0.704	0.742	0.770	0.412	0.755	0.607
BiRNN	0.820	0.860	0.732	0.711	0.726	0.857	0.446	0.786	0.647
RNN	0.851	0.890	0.758	0.743	0.762	0.840	0.499	0.799	0.665
Seq2Seq	0.821	0.866	0.728	0.709	0.730	0.834	0.435	0.779	0.637
CNN	0.899	0.928	0.800	0.783	0.784	0.901	0.591	0.838	0.722
ConvMixer64	0.891	0.923	0.760	0.783	0.934	0.626	0.576	0.749	0.599
DSConv	0.849	0.889	0.746	0.717	0.719	0.915	0.483	0.805	0.674
LSTMCNN	0.978	0.984	0.913	0.910	0.916	0.934	0.822	0.925	0.861
MobileNetV2	0.958	0.968	0.891	0.895	0.938	0.866	0.782	0.901	0.819
ResNet18	**1.000**	**1.000**	**0.998**	**0.998**	**0.999**	0.997	**0.995**	**0.998**	**0.996**
ResNet50	**1.000**	**1.000**	0.996	0.996	0.998	0.996	0.993	0.997	0.994
SCAResNet18	**1.000**	**1.000**	0.997	0.997	0.997	**0.998**	0.994	0.997	0.995
DeepsmirUD-all	0.998	0.999	0.978	0.978	0.982	0.980	0.956	0.981	0.963
DeepsmirUD-top	**1.000**	**1.000**	**0.998**	**0.998**	0.998	**0.998**	**0.995**	**0.998**	**0.996**

Table S2. Prediction performance evaluation on TestSim. DeepsmirUD-all is the ensemble model of 12 individual models and DeepsmirUD-top is the ensemble model of the top best-performing individual models (see *Materials and methods*).

Method	AUC	AUCPR	ACC	bACC	Precision	Recall	MCC	F1score	Jaccard
AlexNet	0.881	0.929	0.786	0.758	0.798	0.877	0.538	0.835	0.717
BiRNN	0.802	0.853	0.779	0.729	0.760	0.938	0.524	0.840	0.724
RNN	0.880	0.929	0.809	0.781	0.811	0.901	0.588	0.854	0.745
Seq2Seq	0.870	0.926	0.756	0.699	0.738	0.938	0.472	0.826	0.704
CNN	0.928	0.957	0.817	0.787	0.813	0.914	0.605	0.860	0.755
ConvMixer64	0.925	0.958	0.863	0.874	0.944	0.827	0.729	0.882	0.788
DSConv	0.910	0.947	0.794	0.753	0.781	0.926	0.555	0.847	0.735
LSTMCNN	0.980	0.989	0.924	0.919	0.938	0.938	0.838	0.938	0.884
MobileNetV2	0.935	0.954	0.893	0.887	0.914	0.914	0.774	0.914	0.841
ResNet18	**0.997**	**0.998**	**0.969**	**0.968**	**0.975**	**0.975**	**0.935**	**0.975**	**0.952**
ResNet50	0.984	0.993	0.931	0.933	0.962	0.926	0.857	0.943	0.893
SCAResNet18	0.987	0.992	0.947	0.941	0.951	0.963	0.886	0.957	0.918
DeepsmirUD-all	0.969	0.983	0.954	0.951	0.963	0.963	0.903	0.963	0.929
DeepsmirUD-top	0.984	0.992	**0.969**	**0.968**	**0.975**	**0.975**	**0.935**	**0.975**	**0.952**

Table S3. Prediction performance evaluation on TestRptMIR. DeepsmirUD-all is the ensemble model of 12 individual models and DeepsmirUD-top is the ensemble model of the top best-performing individual models (see *Materials and methods*).

Method	AUC	AUCPR	ACC	bACC	Precision	Recall	MCC	F1score	Jaccard
AlexNet	0.864	0.872	0.745	0.747	0.697	0.852	0.505	0.767	0.622
BiRNN	0.821	0.848	0.691	0.694	0.639	0.852	0.407	0.730	0.575
RNN	0.868	0.887	0.691	0.692	0.656	0.778	0.390	0.712	0.553
Seq2Seq	0.888	0.901	0.764	0.767	0.684	**0.963**	0.578	0.800	0.667
CNN	0.878	0.885	0.745	0.747	0.710	0.815	0.497	0.759	0.611
ConvMixer64	0.861	0.877	0.727	0.726	0.773	0.630	0.460	0.694	0.531
DSConv	0.832	0.866	0.691	0.695	0.625	0.926	0.438	0.746	0.595
LSTMCNN	0.918	0.894	**0.855**	0.855	**0.828**	0.889	0.711	0.857	0.750
MobileNetV2	0.888	0.891	0.782	0.782	0.778	0.778	0.563	0.778	0.636
ResNet18	0.836	0.836	0.745	0.745	0.760	0.704	0.491	0.731	0.576
ResNet50	0.807	0.813	0.673	0.673	0.655	0.704	0.347	0.679	0.514
SCAResNet18	0.869	0.850	0.818	0.819	0.774	0.889	0.644	0.828	0.706
DeepsmirUD-all	0.909	0.917	0.764	0.765	0.733	0.815	0.531	0.772	0.629
DeepsmirUD-top	**0.930**	**0.932**	**0.855**	**0.856**	0.788	**0.963**	**0.728**	**0.867**	**0.765**

Table S4. Prediction performance evaluation on TestRptSM. DeepsmirUD-all is the ensemble model of 12 individual models and DeepsmirUD-top is the ensemble model of the top best-performing individual models (see *Materials and methods*).

Method	AUC	AUCPR	ACC	bACC	Precision	Recall	MCC	F1score	Jaccard
AlexNet	0.703	0.749	0.617	0.617	0.600	0.700	0.237	0.646	0.477
BiRNN	0.750	0.783	0.633	0.633	0.595	0.833	0.291	0.694	0.532
RNN	0.761	0.798	0.633	0.633	0.611	0.733	0.272	0.667	0.500
Seq2Seq	0.733	0.771	0.667	0.667	0.619	**0.867**	0.364	0.722	0.565
CNN	0.744	0.778	0.633	0.633	0.605	0.767	0.277	0.676	0.511
ConvMixer64	0.766	0.772	0.717	0.717	**0.783**	0.600	0.446	0.679	0.514
DSConv	0.772	0.811	0.650	0.650	0.605	**0.867**	0.333	0.712	0.553
LSTMCNN	0.774	0.792	0.683	0.683	0.641	0.833	0.384	0.725	0.568
MobileNetV2	0.737	0.741	0.717	0.717	0.686	0.800	0.439	0.738	0.585
ResNet18	0.683	0.709	0.650	0.650	0.636	0.700	0.302	0.667	0.500
ResNet50	0.788	0.700	0.733	0.733	0.706	0.800	0.471	0.750	0.600
SCAResNet18	0.684	0.668	0.617	0.617	0.590	0.767	0.245	0.667	0.500
DeepsmirUD-all	0.776	0.797	0.683	0.683	0.649	0.800	0.377	0.716	0.558
DeepsmirUD-top	**0.807**	**0.814**	**0.767**	**0.767**	0.750	0.800	**0.535**	**0.774**	**0.632**

Tables S5-14. Supplementary_Tables_5-14.xlsx

Table S15. Supplementary_Table_15.xlsx

Tables S16-23. Supplementary_Tables_16-23.xlsx

It is noted that the three table documents are made publicly available and can be found at https://github.com/2003100127/deepsmirud and https://rujinlong.github.io/deepsmirud/.

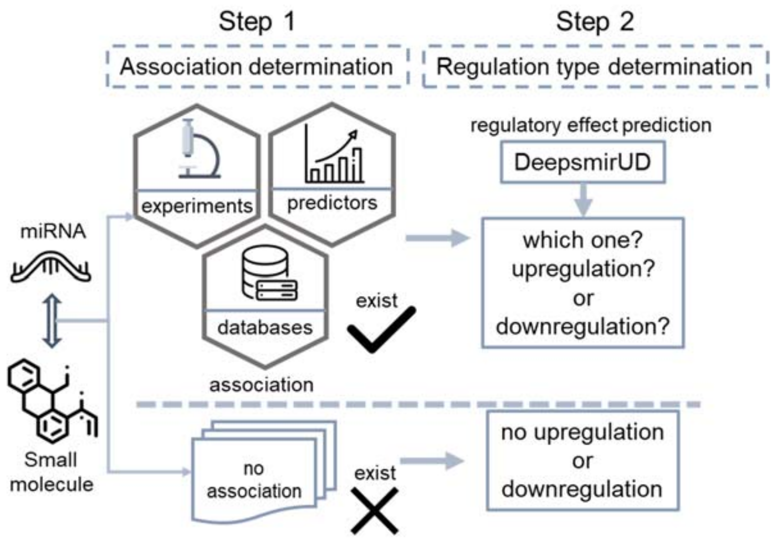

Figure S1. Illustration of the regulatory effect prediction by DeepsmirUD. The two-step pipeline shows the difference between existing predictors and DeepsmirUD. In a word, the association determination precedes the regulation type determination. Starting from a SM-miR pair with an unknown association, the first step of the pipeline will be used to infer the existence of an association between them by existing experiments, predictors (e.g., HSSMMA [23], PSRR [27], TLHNSMMA [74], BNNRSMMA [75], and EKRRSMMA [76]), or databases. After then, this pair is taken as input to DeepsmirUD for regulation type determination if this pair is determined as associating; otherwise, there is no upregulation or downregulation relation between them.

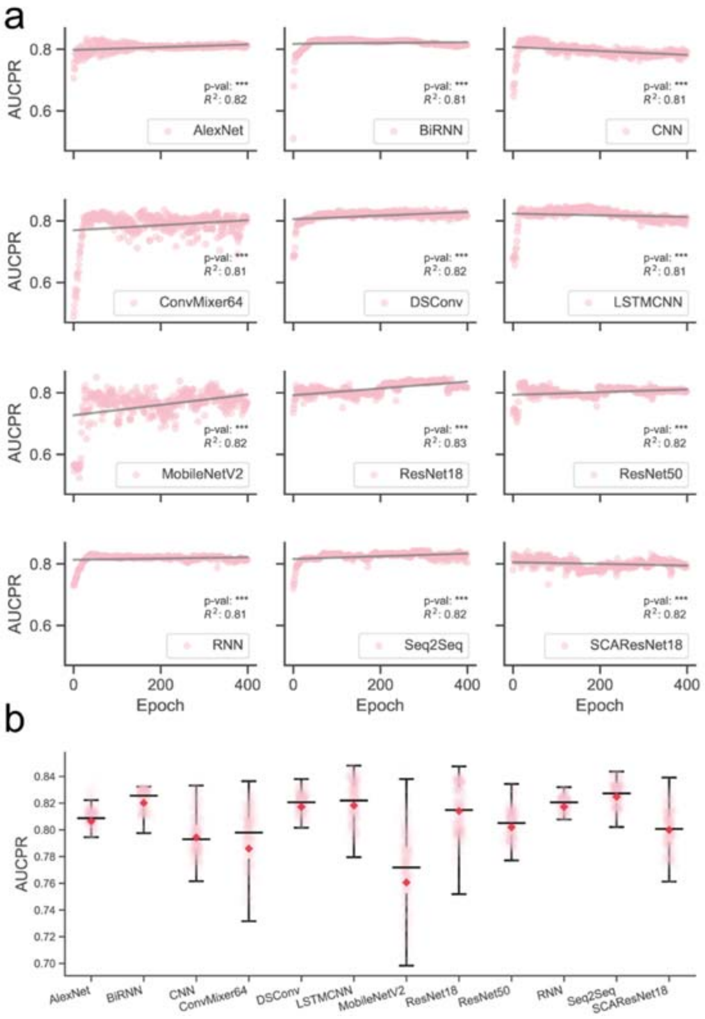

Figure S2. Full-scale AUCPR performance examination of deep learning algorithms on Test over training epochs. a. Landscapes of AUCPR variations. b. Boxplot of AUCPR values. The *p*-val symbols show the statistical significance based on the T-test and *** represents the statistically significant difference. R^2 represents r-squared values. The red dots in the boxplots represent the average prediction values.

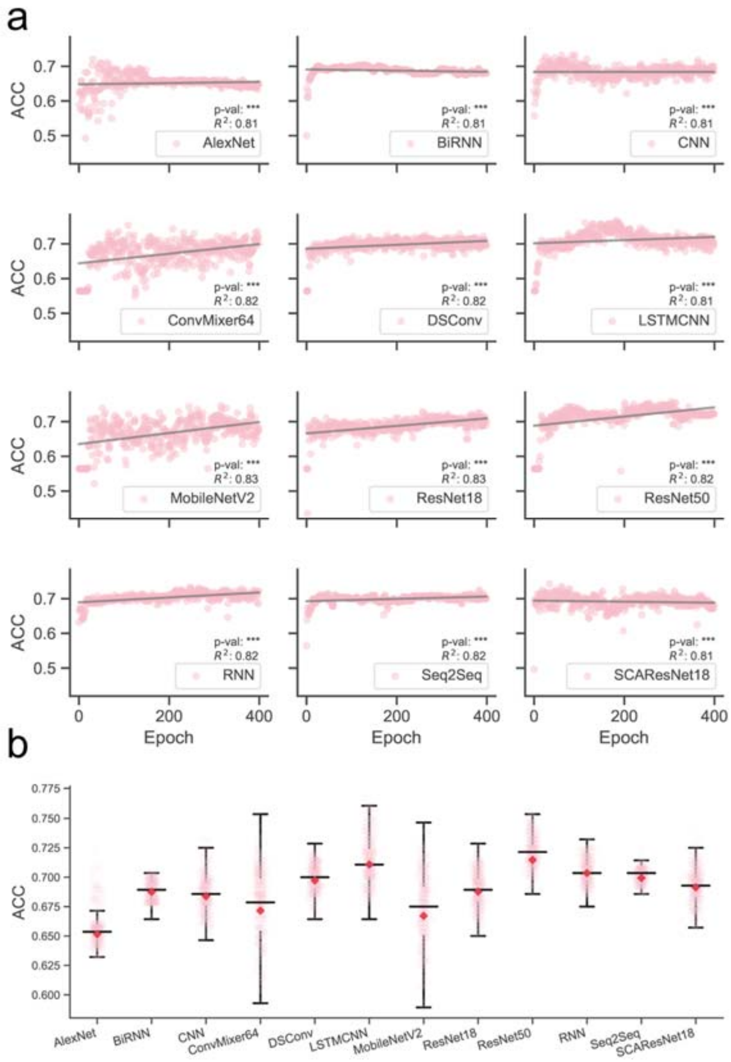

Figure S3. Full-scale ACC performance examination of deep learning algorithms on Test over training epochs. a. Landscapes of ACC variations. b. Boxplot of ACC values. The *p*-val symbols show the statistical significance based on the T-test and *** represents the statistically significant difference. R^2 represents r-squared values. The red dots in the boxplots represent the average prediction values.

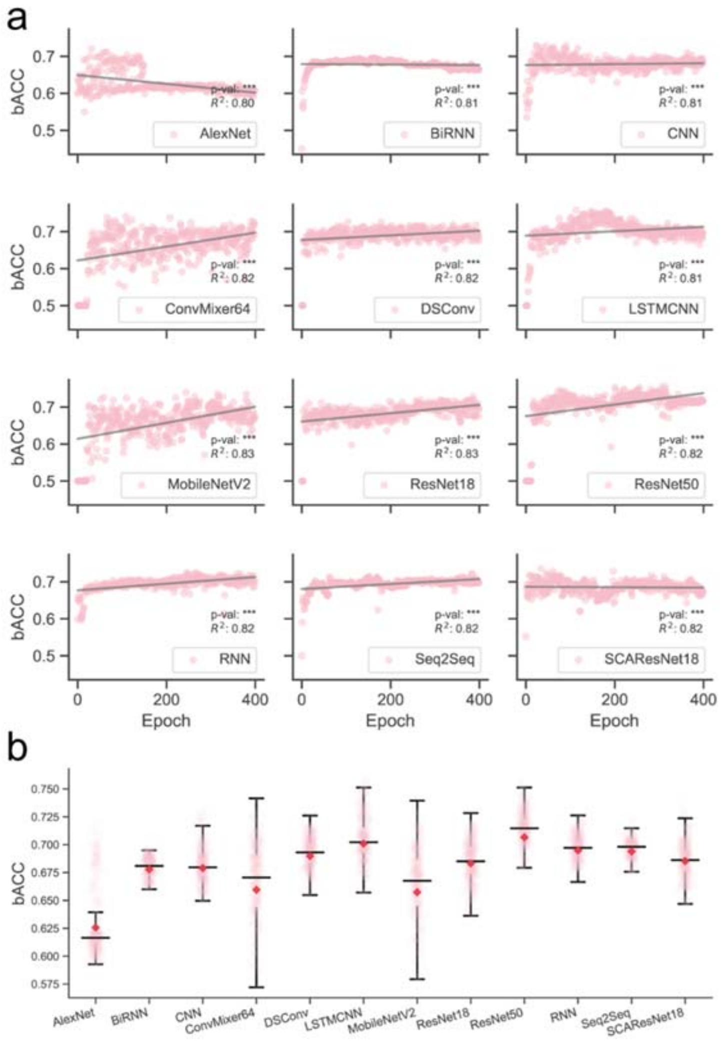

Figure S4. Full-scale bACC performance examination of deep learning algorithms on Test over training epochs. a. Landscapes of bACC variations. b. Boxplot of bACC values. The *p*-val symbols show the statistical significance based on the T-test and *** represents the statistically significant difference. R^2 represents r-squared values. The red dots in the boxplots represent the average prediction values.

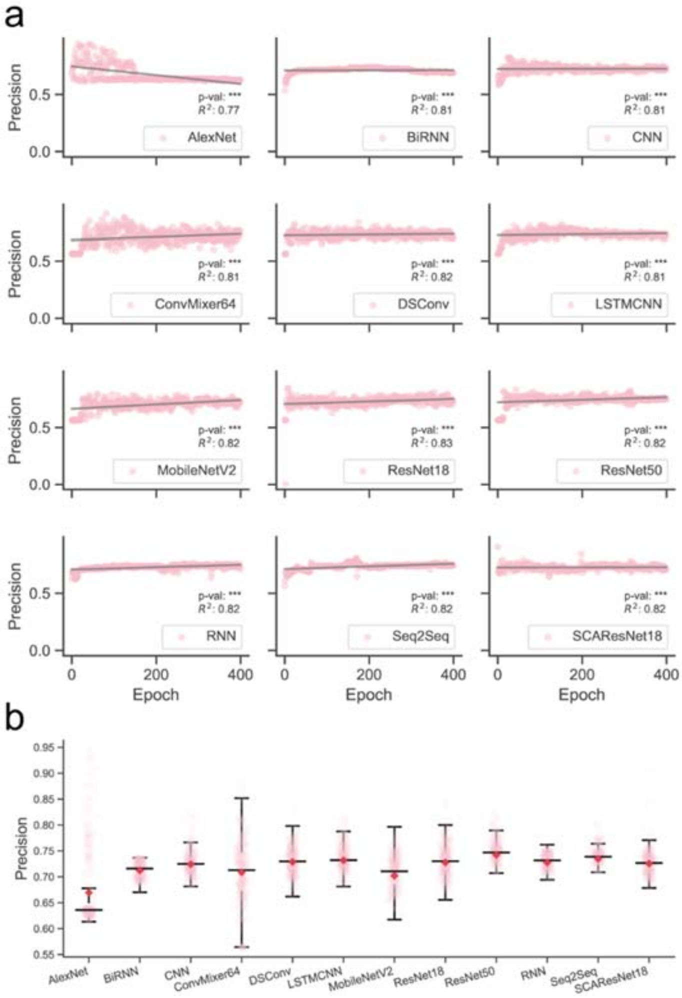

Figure S5. Full-scale precision performance examination of deep learning algorithms on Test over training epochs. a. Landscapes of precision variations. b. Boxplot of precision values. The *p*-val symbols show the statistical significance based on the T-test and *** represents the statistically significant difference. R^2 represents r-squared values. The red dots in the boxplots represent the average prediction values.

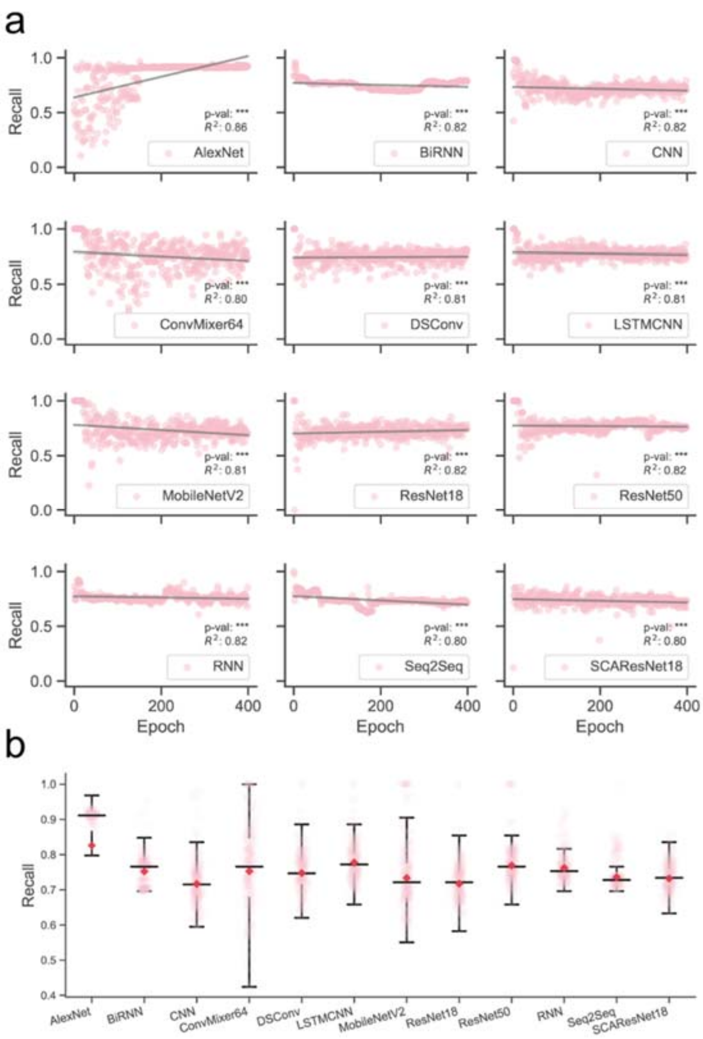

Figure S6. Full-scale recall performance examination of deep learning algorithms on Test over training epochs. a. Landscapes of recall variations. b. Boxplot of recall values. The *p*-val symbols show the statistical significance based on the T-test and *** represents the statistically significant difference. R^2 represents r-squared values. The red dots in the boxplots represent the average prediction values.

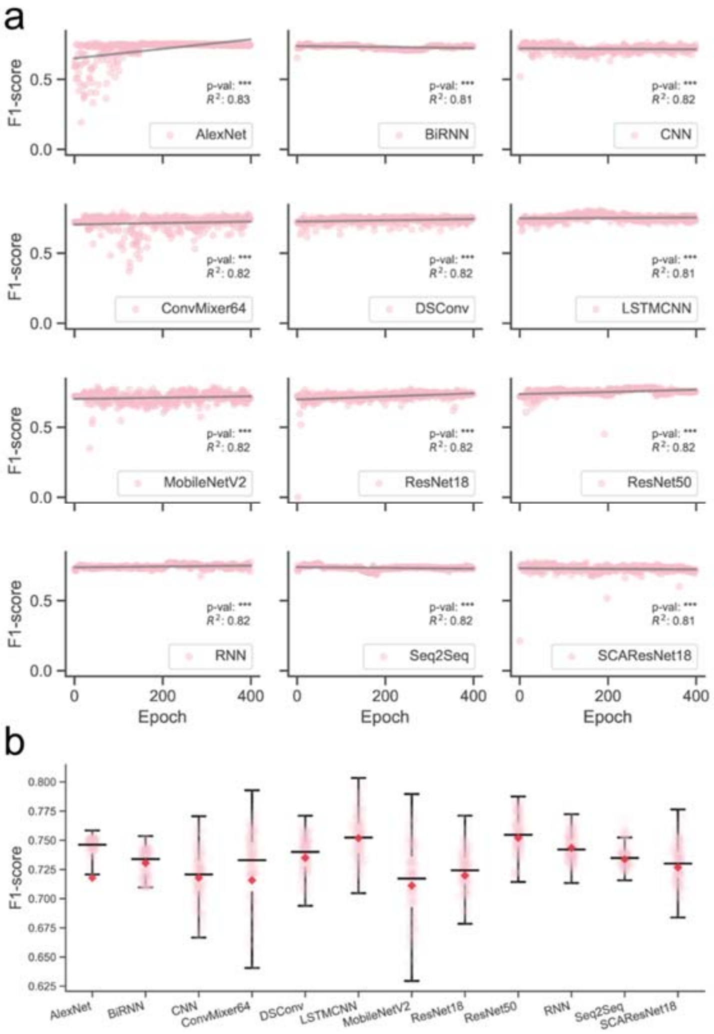

Figure S7. Full-scale F1-score performance examination of deep learning algorithms on Test over training epochs. a. Landscapes of F1-score variations. b. Boxplot of F1-score values. The *p*-val symbols show the statistical significance based on the T-test and *** represents the statistically significant difference. R^2 represents r-squared values. The red dots in the boxplots represent the average prediction values.

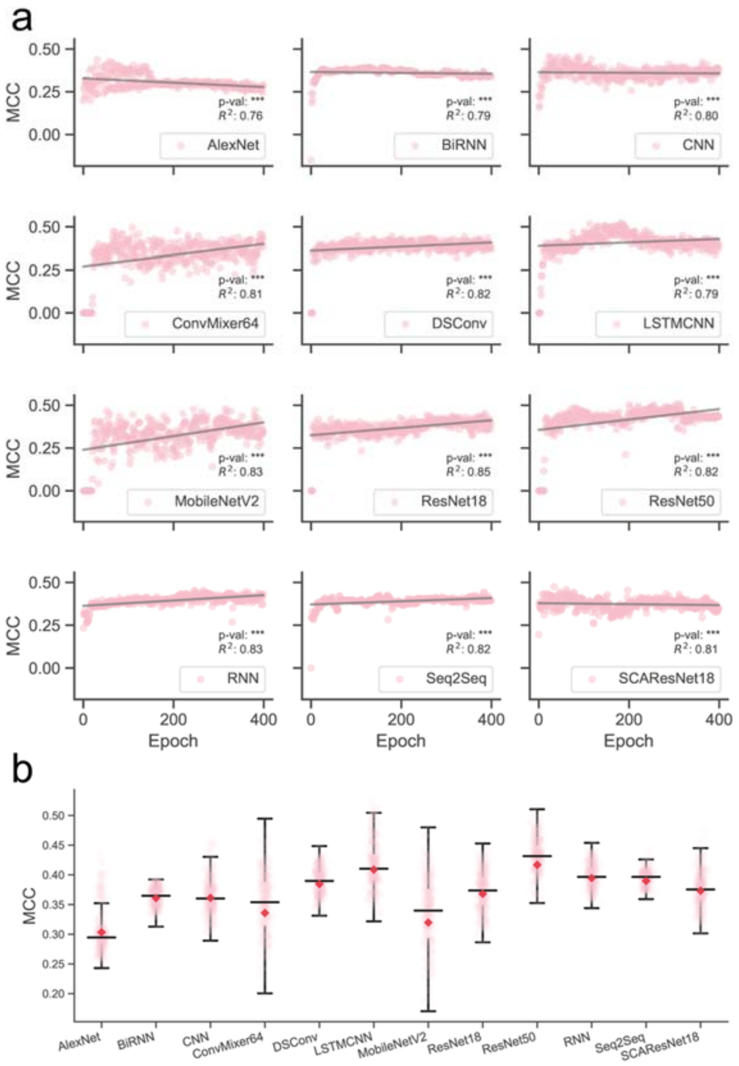

Figure S8. Full-scale MCC performance examination of deep learning algorithms on Test over training epochs. a. Landscapes of MCC variations. b. Boxplot of MCC values. The *p*-val symbols show the statistical significance based on the T-test and *** represents the statistically significant difference. R^2 represents r-squared values. The red dots in the boxplots represent the average prediction values.

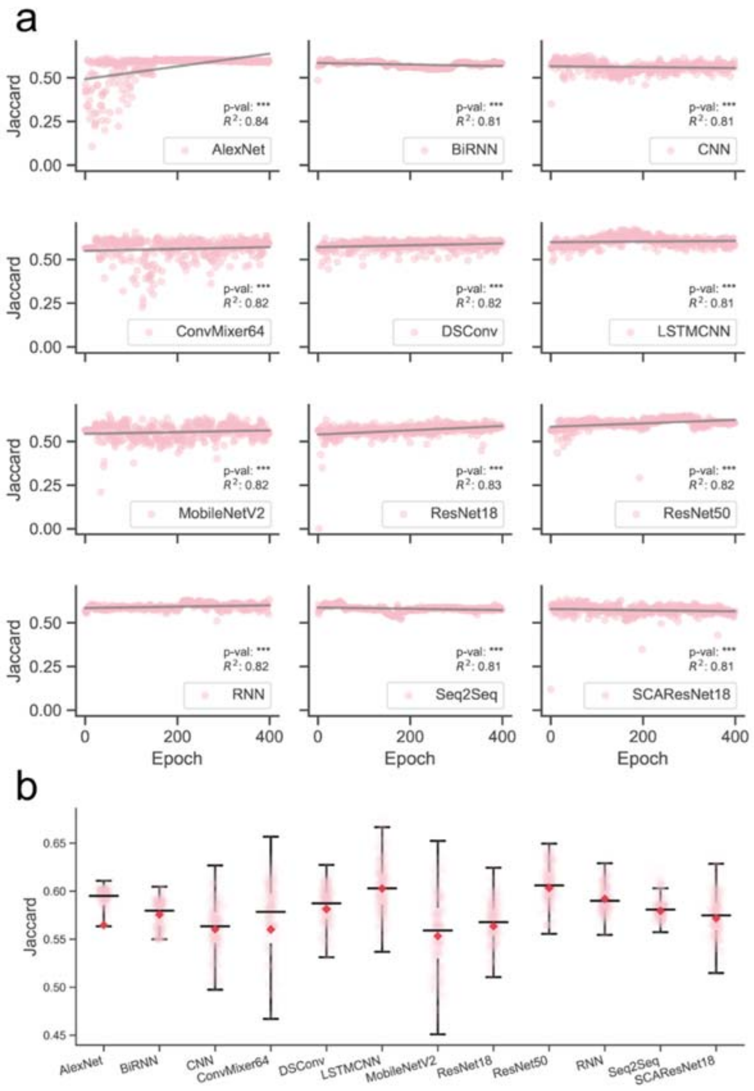

Figure S9. Full-scale Jaccard performance examination of deep learning algorithms on Test over training epochs. a. Landscapes of Jaccard variations. b. Boxplot of Jaccard values. The *p*-val symbols show the statistical significance based on the T-test and *** represents the statistically significant difference. R^2 represents r-squared values. The red dots in the boxplots represent the average prediction values.

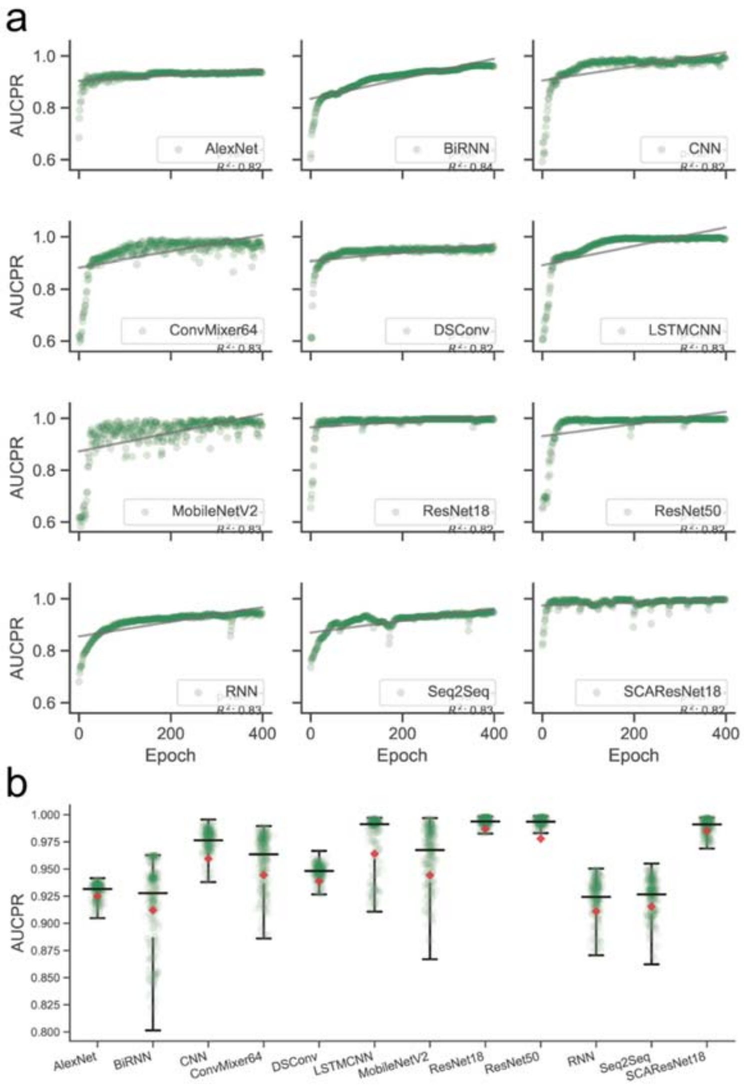

Figure S10. Full-scale AUCPR performance examination of deep learning algorithms on TestSim over training epochs. a. Landscapes of AUCPR variations. b. Boxplot of AUCPR values. The *p*-val symbols show the statistical significance based on the T-test and *** represents the statistically significant difference. R^2 represents r-squared values. The red dots in the boxplots represent the average prediction values.

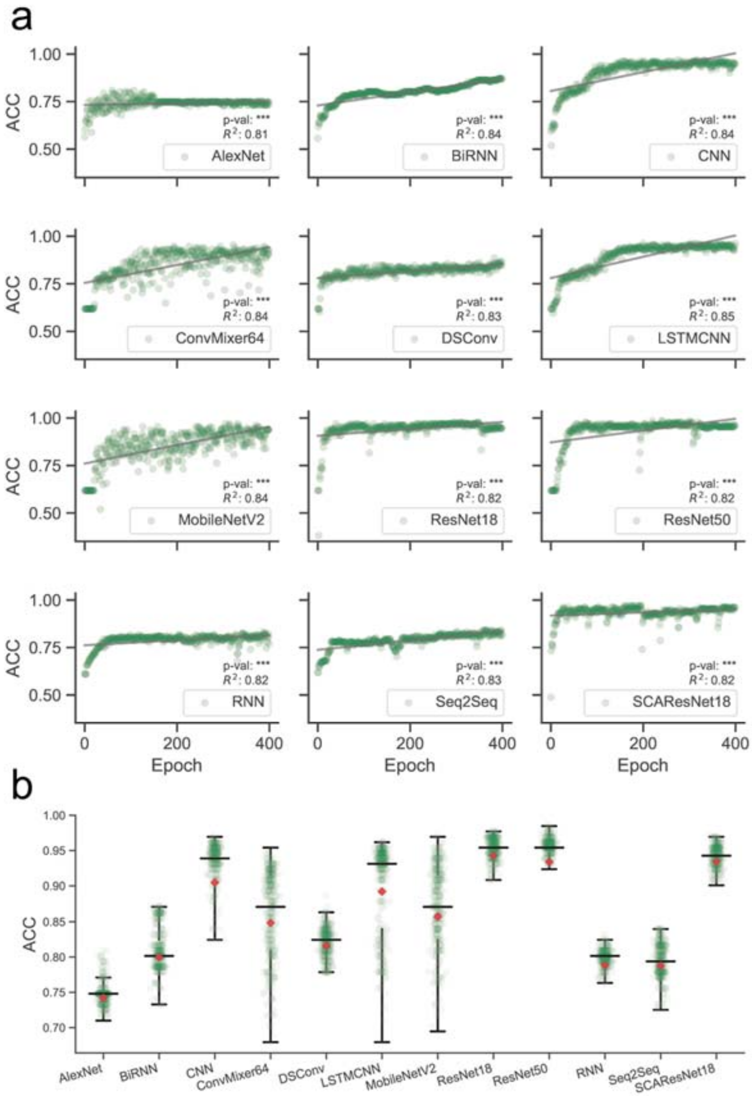

Figure S11. Full-scale ACC performance examination of deep learning algorithms on TestSim over training epochs. a. Landscapes of ACC variations. b. Boxplot of ACC values. The *p*-val symbols show the statistical significance based on the T-test and *** represents the statistically significant difference. R^2 represents r-squared values. The red dots in the boxplots represent the average prediction values.

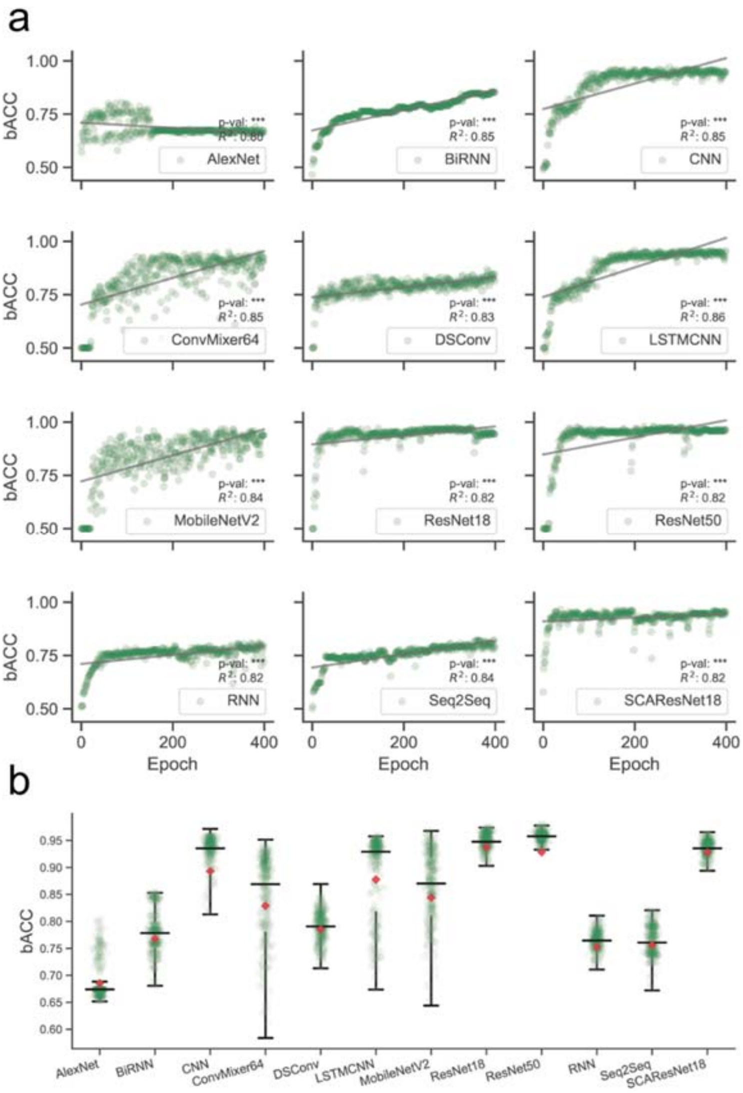

Figure S12. Full-scale bACC performance examination of deep learning algorithms on TestSim over training epochs. a. Landscapes of bACC variations. b. Boxplot of bACC values. The *p*-val symbols show the statistical significance based on the T-test and *** represents the statistically significant difference. R^2 represents r-squared values. The red dots in the boxplots represent the average prediction values.

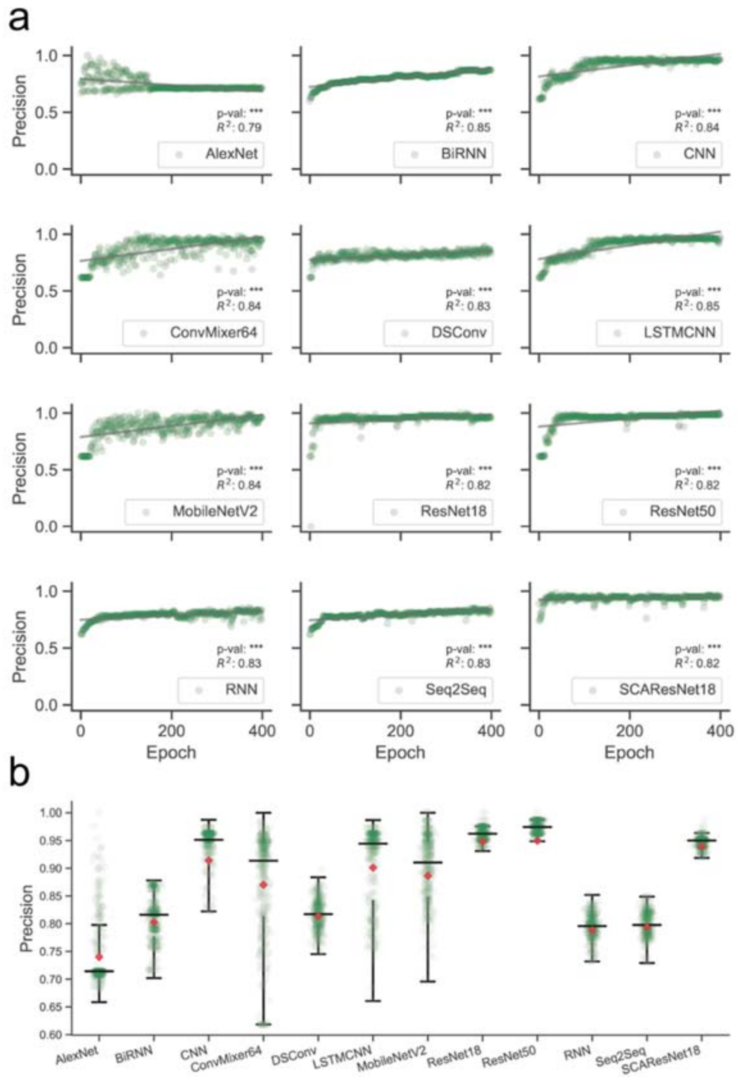

Figure S13. Full-scale precision performance examination of deep learning algorithms on TestSim over training epochs. a. Landscapes of precision variations. b. Boxplot of precision values. The *p*-val symbols show the statistical significance based on the T-test and *** represents the statistically significant difference. R^2 represents r-squared values. The red dots in the boxplots represent the average prediction values.

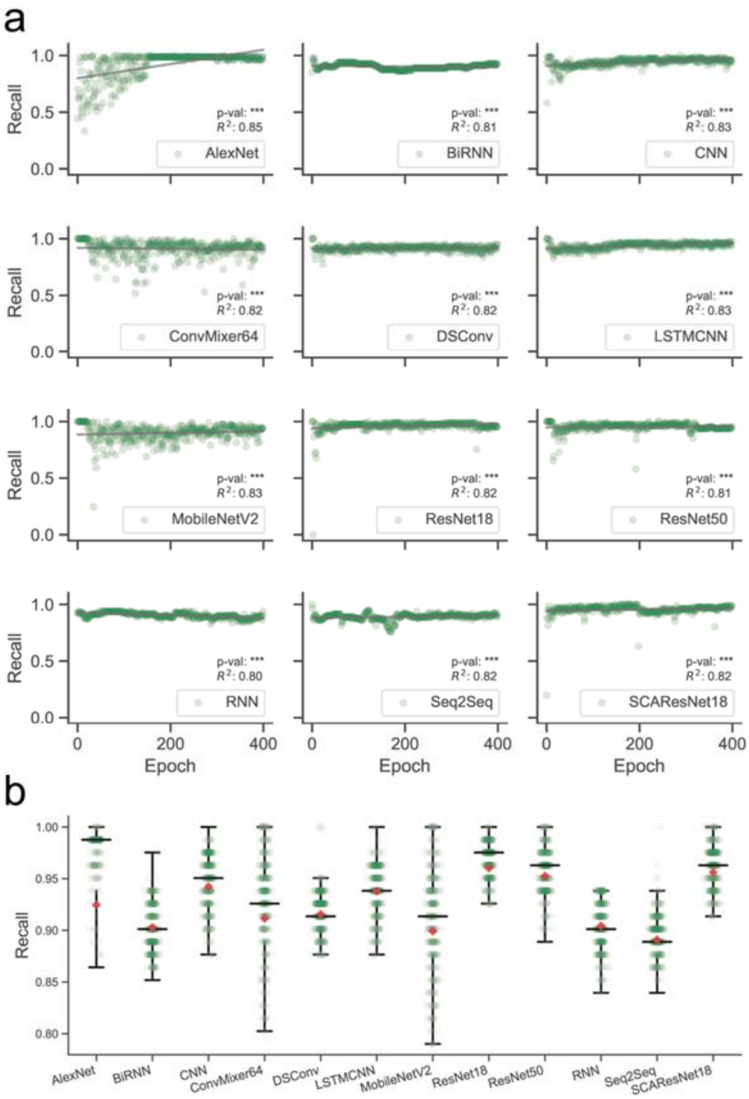

Figure S14. Full-scale recall performance examination of deep learning algorithms on TestSim over training epochs. a. Landscapes of recall variations. b. Boxplot of recall values. The *p*-val symbols show the statistical significance based on the T-test and *** represents the statistically significant difference. R^2 represents r-squared values. The red dots in the boxplots represent the average prediction values.

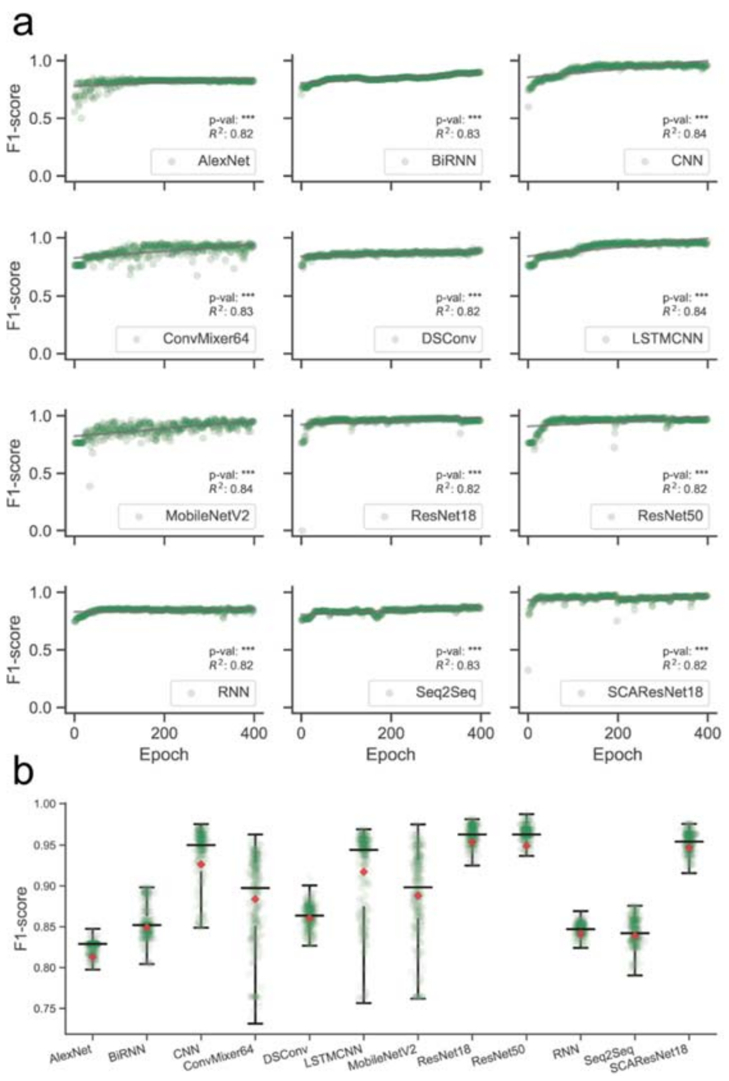

Figure S15. Full-scale F1-score performance examination of deep learning algorithms on TestSim over training epochs. a. Landscapes of F1-score variations. b. Boxplot of F1-score values. The *p*-val symbols show the statistical significance based on the T-test and *** represents the statistically significant difference. R^2 represents r-squared values. The red dots in the boxplots represent the average prediction values.

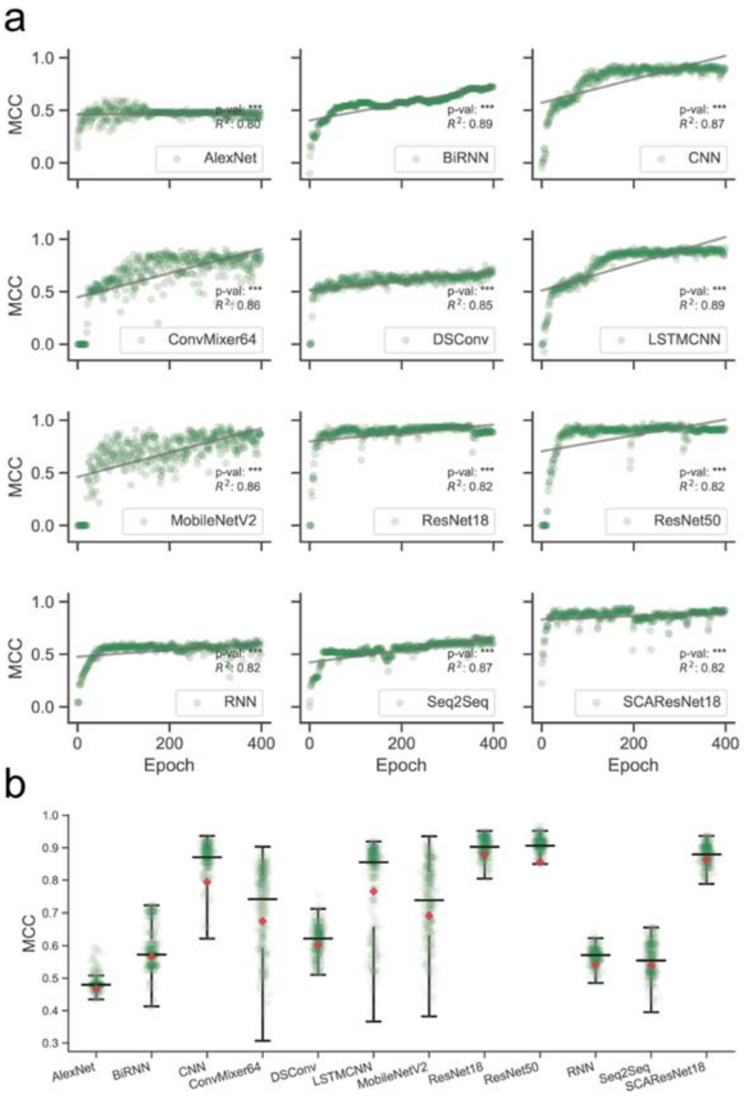

Figure S16. Full-scale MCC performance examination of deep learning algorithms on TestSim over training epochs. a. Landscapes of MCC variations. b. Boxplot of MCC values. The *p*-val symbols show the statistical significance based on the T-test and *** represents the statistically significant difference. R^2 represents r-squared values. The red dots in the boxplots represent the average prediction values.

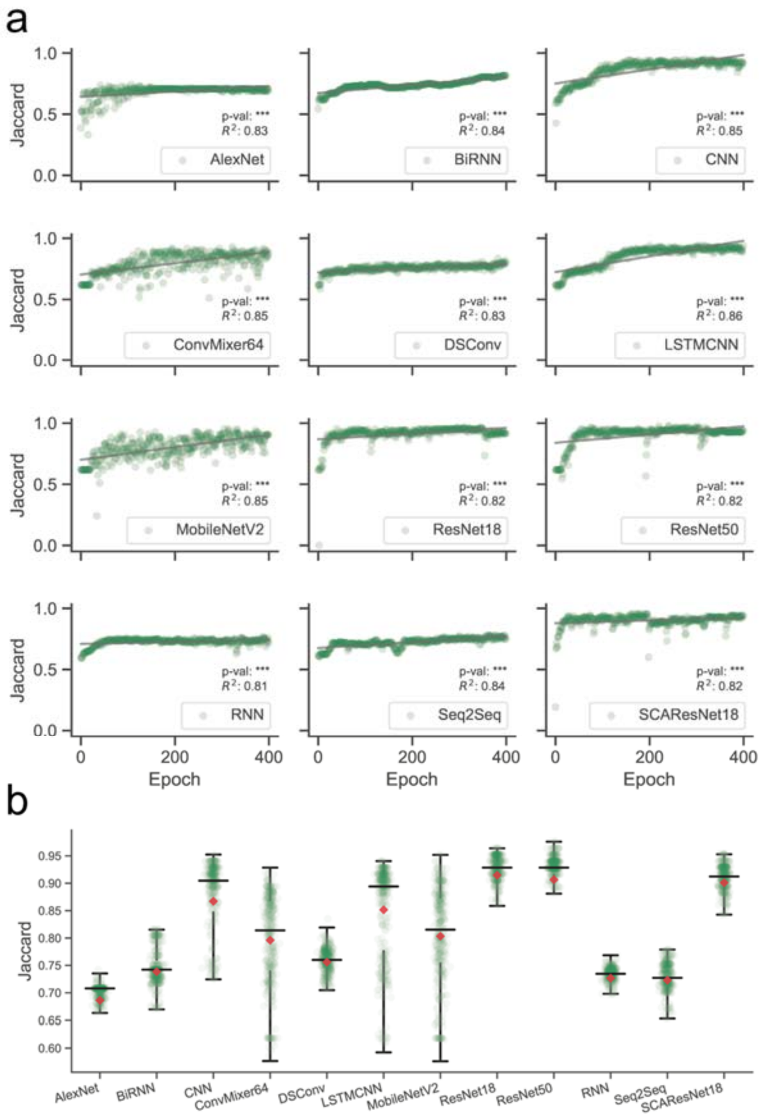

Figure S17. Full-scale Jaccard performance examination of deep learning algorithms on TestSim over training epochs. a. Landscapes of Jaccard variations. b. Boxplot of Jaccard values. The *p*-val symbols show the statistical significance based on the T-test and *** represents the statistically significant difference. R^2 represents r-squared values. The red dots in the boxplots represent the average prediction values.

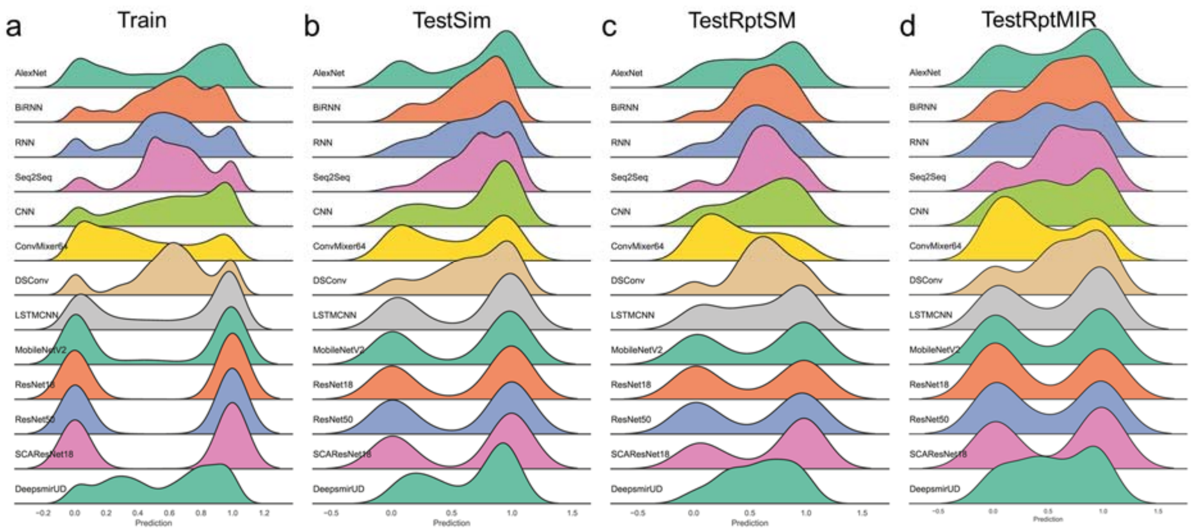

Figure S18. Ridge plot of prediction values (i.e., regulatory effects) on Train, TestSim, TestRptSM, and TestRptMIR.

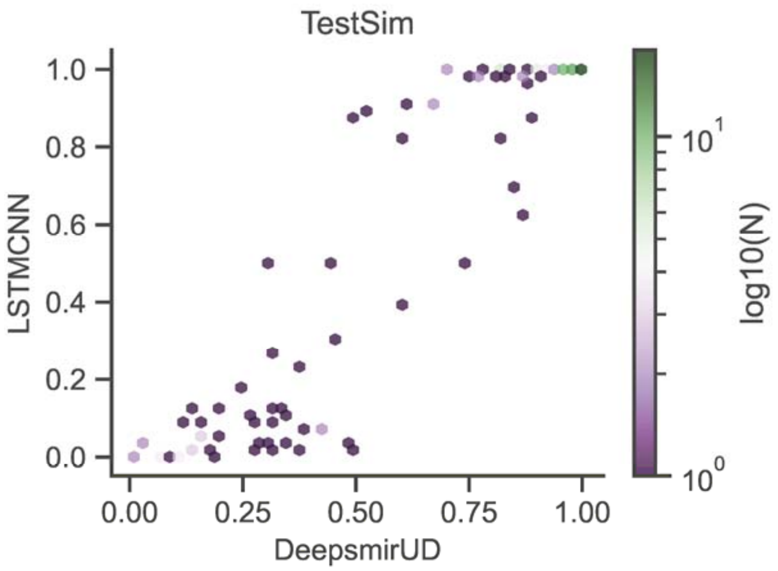

Figure S19. Hexagonal binned plot of comparison between LSTMCNN and DeepsmirUD predictions on TestSim.
